# Quantitative analysis of human adult pancreatic histology reveals separate fatty and fibrotic phenotypes in type 2 diabetes

**DOI:** 10.1007/s00125-025-06547-8

**Published:** 2025-09-24

**Authors:** Nicola J. Dyson, Nicole Kattner, Yara Al-Selwi, Minna Honkanen-Scott, Morgan F. Shaw, Caitlin A. Brack, Rowen Coulthard, Christine S. Flaxman, Sarah J. Richardson, James A. M. Shaw

**Affiliations:** 1 Translational and Clinical Research Institute, Newcastle University, Newcastle upon Tyne, UK; 2 Department of Cellular Pathology, Royal Victoria Infirmary, Newcastle upon Tyne Hospitals NHS Trust, Newcastle upon Tyne, UK; 3 Islet Biology Exeter (IBEx), Exeter Centre of Excellence for Diabetes Research (EXCEED), Department of Clinical and Biomedical Sciences, University of Exeter Medical School, Exeter, UK; 4 Institute of Transplantation, Freeman Hospital, Newcastle upon Tyne Hospitals NHS Foundation Trust, Newcastle upon Tyne, UK

**Keywords:** Adipocyte, Fibrosis, Histology, Lipid droplet, Pancreas, Type 2 diabetes

## Abstract

**Aims/hypothesis:**

The role of intra-pancreatic lipid and collagen in type 2 diabetes pathogenesis remains unclear. We sought to examine this in pancreases from organ donors with and without diabetes.

**Methods:**

Tissue biopsies from 36 adult donor pancreases with/without type 2 diabetes were collected from 16 anatomically defined regions, with H&E, Sirius Red Fast Green and chromogranin A immunohistochemical staining and quantification performed. Intracellular lipid droplet area was quantified using transmission electron microscopy in acinar, islet endocrine, beta and alpha cells identified through ultrastructural morphology.

**Results:**

Increasing adipocyte proportional area was associated with increasing pancreas donor BMI (*r*=0.385, *p*=0.021), decreased acinar area (*r*=−0.762, *p*<0.001) and increased endocrine mass (*r*=0.749, *p*<0.001). Fibrosis was not associated with BMI, acinar area or endocrine mass. Type 2 diabetes was associated with decreased islet circularity and reduced beta:alpha cell ratio but endocrine mass was not affected. Adipocyte and fibrosis proportional areas were highest in donors with diabetes but not associated with each other. Pancreases with high fat and those with high fibrosis (>40% proportional area) appeared to form two separate subgroups. All donors with insulin-treated diabetes had a high collagen proportional area. Fibrosis but not adipocytosis was associated with decreased beta:alpha cell ratio. There was an inverse relationship between pancreatic adipocytosis and intra-acinar cell lipid content (*r*=−0.490, *p*=0.003), with the lowest levels seen in type 2 diabetes. Beta cell lipid content was associated with BMI but not type 2 diabetes.

**Conclusions/interpretation:**

Systematic human pancreatic analysis revealed two separate type 2 diabetes phenotypes: fatty, associated with central obesity; and fibrotic, associated with reduced beta cell mass without central obesity. This suggests distinct underlying pathogenic mechanisms and has potential for developing personalised disease-modifying therapeutics.

**Graphical Abstract:**

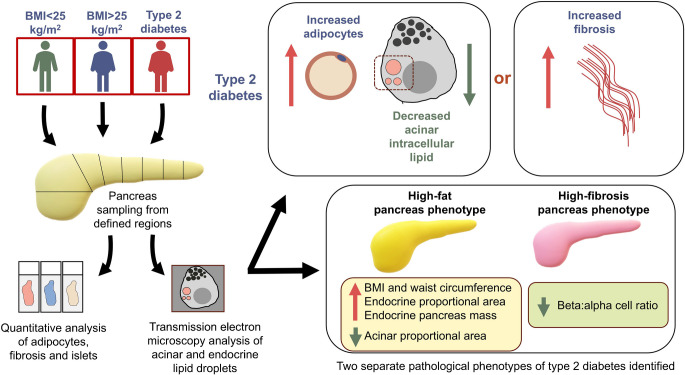

**Supplementary Information:**

The online version contains peer-reviewed but unedited supplementary material available at 10.1007/s00125-025-06547-8.


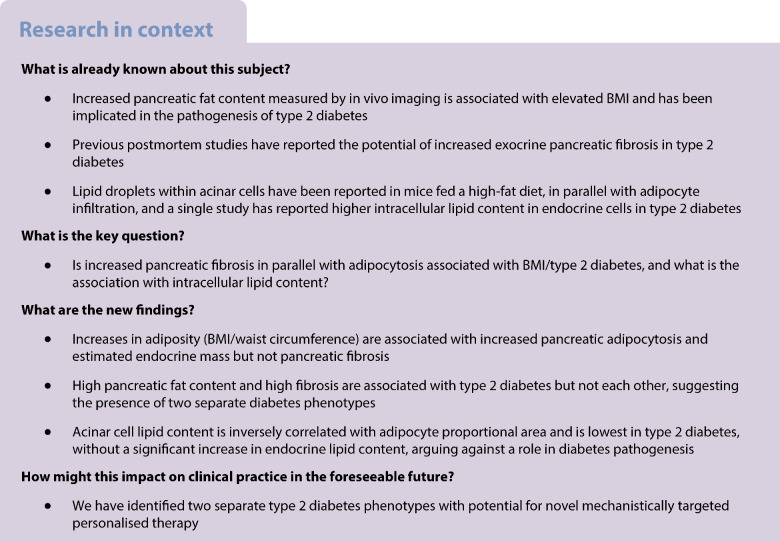


## Introduction

Type 2 diabetes is a growing global health burden, with obesity established as a major risk factor underlying its development [1, 2]. Excess ectopic fat in the pancreas and liver appear to confer increased type 2 diabetes risk [3]. Pancreatic fat content measured by MRI has been shown to be positively associated with BMI, waist circumference (WC) [4] and insulin resistance [5]. Increased pancreatic fat in type 2 diabetes has been described in comparison with weight-matched control groups [6], with reduction accompanied by increased pancreatic volume and border ‘smoothness’ in parallel with diabetes remission induced through weight loss [7].

In the pancreas, fat is present as adipocytes surrounding (extralobular) and within the lobes (intralobular) of the pancreatic parenchyma, and as intracellular lipid droplets (LDs) located within acinar and endocrine cells. Adipocytes and LDs have wider metabolic functions beyond lipid storage [8, 9]. Recent evidence suggests differences between pancreatic endocrine and exocrine LD accumulation patterns in individuals with type 2 diabetes vs control individuals [10], but associations between intra-pancreatic LDs, adipocytes and type 2 diabetes have not been fully elucidated.

Intra-pancreatic adipocytes and, in some studies, collagen have been reported to increase with age [11–13]. Collagen is a normal structural constituent of the islet basement membrane [14] also bounding acinar lobules and endocrine vasculature [15]. Chronic inflammatory injury can lead to excess collagen deposition and fibrosis [16]. This is driven by pancreatic stellate cells, which are activated in response to inflammation and produce collagen and other extracellular matrix components [17]. In metabolic dysfunction-associated steatotic liver disease (MASLD), hepatic steatosis results in a proinflammatory environment from which fibrosis and, eventually, cirrhosis can develop [18]. Fatty and fibrotic replacement of acinar tissue is evident in pancreatogenic diseases, including chronic pancreatitis and cystic fibrosis [19]. However, while increased pancreatic fibrosis has been previously reported in type 2 diabetes [20], the pathophysiology and relationship between pancreatic fat deposition and fibrosis are not fully understood. A recent analysis in human donor pancreases reported increased intralobular collagen deposition in type 2 diabetes without correlations with age or BMI in donors without diabetes [13]. In contrast, pancreatic adiposity increased with age and BMI but was not higher in donors with diabetes [13].

We aimed to characterise the relationship between lipid accumulation and collagen deposition in a cohort of human pancreases from deceased organ donors with normal body weight, overweight without diabetes and overweight with type 2 diabetes using image analysis pipelines and ultrastructural examination.

## Methods

### Donor selection and sample collection

The study was undertaken in a cohort of 36 donors selected from a larger collection of whole pancreases retrieved from deceased organ donors in the UK within the Quality in Organ Donation Medical Research Council Expand Tissue Bank (QUOD-PANC). The cohort comprised 12 donors with a diagnosis of type 2 diabetes and with BMI >25 kg/m^2^; 12 donors with BMI >25 kg/m^2^ and no history of diabetes with HbA_1c_ during terminal admission <42 mmol/mol (6.0%); and 12 donors with BMI <25 kg/m^2^ and no history of diabetes with HbA_1c_ during terminal admission <42 mmol/mol (6.0%) (electronic supplementary material [ESM] Table 1). Donors without diabetes with BMI >25 kg/m^2^ were selected to enable BMI similarity to those with diabetes as much as possible, and donor ages in all three subgroups were selected to be as similar as possible.

Pancreases were retrieved by the UK National Organ Retrieval Service, following written donor relative consent, in compliance with the 2004 UK Human Tissue Act. Tissue was collected, processed, stored and analysed under ethical approvals 05/MRE09/48 and 16NE0230. Donor information, including sex and ethnicity data (ESM Table 1), was obtained from UK NHS Blood and Transplant, medical records, donor families and physical examination. Ethnicity, where available, was recorded as ‘white’ in all cases. No socioeconomic data were available. Donor ages were reflective of the UK adult population (range 18–78 years).

**Fig. 1:**
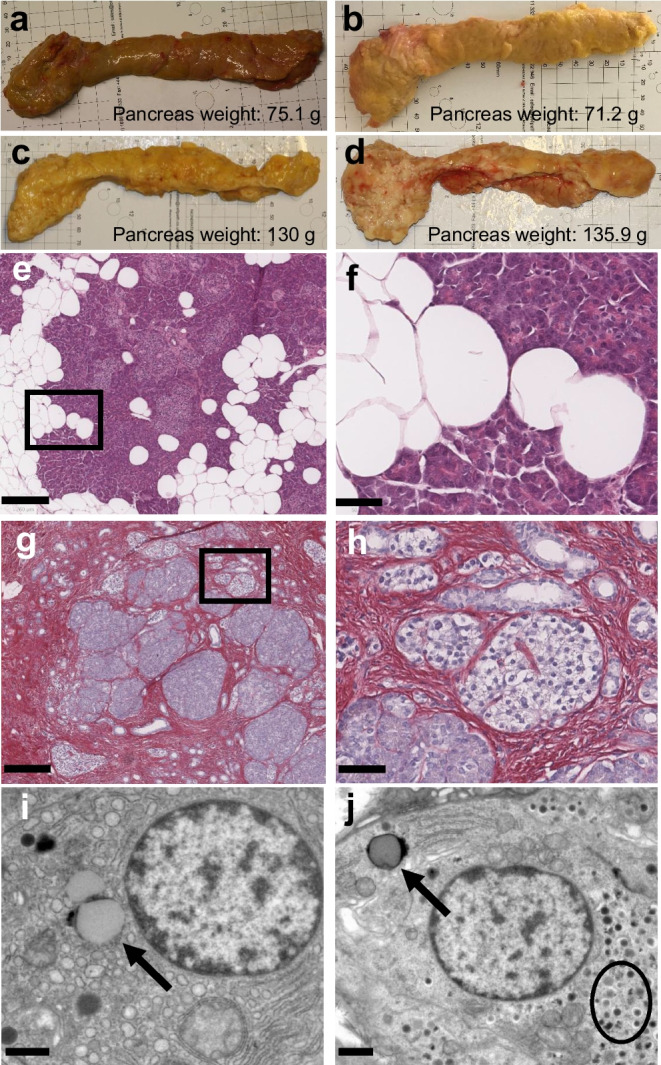
Macroscopic, histological and ultrastructural assessment of the pancreas. (**a**) Representative image of a pancreas from a donor without diabetes without visible infiltrating fat on macroscopic assessment. (**b**) Representative image of a pancreas from a donor with type 2 diabetes with macroscopic infiltrating fat. (**c**) Representative image of a pancreas from a ‘high-fat’ donor with type 2 diabetes with infiltrating fat and a characteristic yellow macroscopic appearance. (**d**) Representative image of a pancreas from a ‘high-fibrosis’ donor with type 2 diabetes with macroscopic infiltrating fat. (**e**, **f**) Example H&E images of adipocytes within pancreatic parenchyma in a ‘high-fat’ donor. (**g**, **h**) Example SRFG images of collagen in a ‘high-fibrosis’ donor. (**i**, **j**) Representative TEM images with arrows indicating intracellular LDs in an acinar cell (**i**) and an endocrine cell (**j**). LDs were identified as rounded vesicles with contents of uniformly moderate electron density, surrounded by a membrane of variable electron density. In islets, beta cells were recognisable by the characteristic ‘halo’ around the insulin granules (circled in **j**) in contrast to the more electron-dense glucagon granules in alpha cells. Pancreas weight is indicated in (**a**–**d**). Scale bars, 250 µm (**e** and **g**), 50 µm (**f** and **h**) and 1 µm (**i** and **j**). Areas within black boxes in (**e**) and (**g**) are shown at higher magnification in (**f**) and (**h**)

Pancreases were flushed with University of Wisconsin solution (Bridge to Life, Wandsworth, UK), then transported in University of Wisconsin solution on ice. Pancreas dissection from spleen, duodenum and extra-pancreatic fat and tissue sampling were performed in a standardised manner as described previously [21, 22] (ESM Fig. 1). Tissue specimens were collected from eight incremental pancreas regions from head to tail denoted P1–P8. Each region was divided into anterior and posterior locations for formalin fixation and paraffin embedding, resulting in 16 different anatomical locations for analysis per donor. Samples for electron microscopy were collected from a subsequent tissue slice from each of the eight anatomical regions. Categorical visual macroscopic assessment of pancreas fat was recorded by the operator dissecting, classified as ‘no fat’; ‘fat surrounding pancreas’; or ‘fat surrounding and infiltrating pancreas’ (Fig. 1).

### Histology

Formalin-fixed tissues were dehydrated, paraffin embedded, sectioned and stained at the NovoPath cellular pathology laboratory (Royal Victoria Infirmary, Newcastle upon Tyne, UK). The 4 µm serial sections were stained with H&E and Sirius Red Fast Green (SRFG) (Atom Scientific, Hyde, UK) using NovoPath optimised protocols [23]. Immunohistochemistry staining for chromogranin A (CGA) with antibody ab15160 (Abcam, Cambridge, UK) was performed on the Discovery autostainer (Roche Diagnostics, Burgess Hill, UK).

Stained slides were digitally scanned (SCN400 slide scanner, Leica Biosystems, Deer Park, IL, USA). The HALO Image Analysis platform with the DenseNet artificial intelligence (AI) v2 plug-in (Indica Labs, Albuquerque, NM, USA) was used for quantitative analysis of collagen (SRFG staining), adipocyte area (H&E staining) and islet numbers and morphometry (CGA staining) (ESM Fig. 1). All 16 regions in each donor were assessed by AI-augmented image analysis. Adipocyte deposition was defined as intralobular or extralobular based on location relative to pancreatic parenchyma. Collagen areas were subdivided by location into acinar, intra-islet or ductal/vessel/other unclassified area. The immediate peri-islet area was further interrogated by defining a region 30 µm in width surrounding each islet and quantifying the percentage of this ‘halo’ comprising collagen. Measurements were normalised to the total tissue area/region under assessment and expressed as percentages. Islet density was measured as mean islet number per mm^2^ of tissue.

AI analysis of intralobular fat was validated by comparison with measurements of the P4 anterior (P4A, body) region carried out manually using QuPath v0.3.2 [24] and ImageJ v2.1.0/1.53c [25] by two separate operators. Both operators’ measurements were highly correlated with those of the AI (Pearson’s *r*, Operator 1: *n*=34 donors [ten type 2 diabetes, 12 BMI >25 kg/m^2^, 12 BMI <25 kg/m^2^], *r*=0.88; *p*<0.0001; Operator 2: *n*=18 donors [six type 2 diabetes, six BMI >25 kg/m^2^, six BMI <25 kg/m^2^], *r*=0.92; *p*<0.0001).

### Transmission electron microscopy

Glutaraldehyde-fixed tissue biopsies from the P4 (body) region of each pancreas were stained with osmium tetroxide and embedded in epoxy resin (TAAB, Aldermaston, UK) followed by post-staining of 70 nm ultrathin sections with uranyl acetate and lead citrate (Leica Microsystems, Milton Keynes, UK). Sections were cut (Ultracut E, Reichert, Vienna, Austria) and mounted on pioloform-coated Ni grids (Gilder Grids, Grantham, UK). Images were acquired on the Hitachi HT7800 120 kV transmission electron microscope (Hitachi High-Technologies, Maidenhead, UK) for analysis. Image acquisition of individual acinar and endocrine cells was performed in a standardised manner as described previously, with 25 acinar and 10–25 endocrine cells assessed per specimen [21, 22].

Intracellular LD area was quantified using transmission electron microscopy (TEM) in acinar, endocrine, beta and alpha cells identified through ultrastructural morphology [22].

### Statistics

Statistical analyses were performed using GraphPad Prism v9 (GraphPad, San Diego, CA, USA) and summarised as mean ± SD. For overall adipocyte and SRFG comparisons with donor characteristics and other AI-derived parameters, the mean of all 16 regions was used. Endocrine mass was estimated by multiplying mean endocrine proportional area (PA) of all 16 regions by post-dissection pancreas weight. P4A region formalin-fixed paraffin-embedded tissue blocks were used for comparisons with ultrastructural data. Comparisons between donor subgroups were performed by unpaired *t* test or one-way ANOVA with Fisher’s least significant difference test for multiple comparisons. Correlations between parameters were analysed using Pearson’s *r*. Contingency analysis was performed using Fisher’s exact test. *k*-means clustering was performed to confirm the groupings of fat and fibrosis subtypes. Statistical significance was classified at *p*<0.05.

## Results

### Donor characteristics

Mean donor age within the overall cohort was 54.0 ± 13.9 years (range 18–78 years), and was comparable between the three subgroups (ESM Table 1, ESM Fig. 2a). There were 15 (42%) male participants, with the sex ratio skewed toward male participants among donors with diabetes and female participants among those without diabetes (ESM Table 1, ESM Fig. 2b). Mean BMI was comparable between those with BMI >25 kg/m^2^ with (33.3 ± 7.9 kg/m^2^) and without (30.8 ± 3.2) diabetes, but was significantly lower in those with normal weight and no diabetes (22.2 ± 2.1 kg/m^2^) (ESM Table 1, ESM Fig. 2c). Donor WC was higher in both groups with BMI >25 kg/m^2^ (ESM Fig. 2d). One donor with type 2 diabetes had previously had a single episode of acute pancreatitis, with no histological evidence of chronic pancreatitis in this or any other donor (ESM Table 1). Glucose-lowering medication was recorded for 11/12 donors with type 2 diabetes: two (18%) were insulin-treated, six (55%) on oral medication and three (27%) on diet alone (ESM Table 1).

### Pancreatic weight and macroscopic fat infiltration

**Fig. 2:**
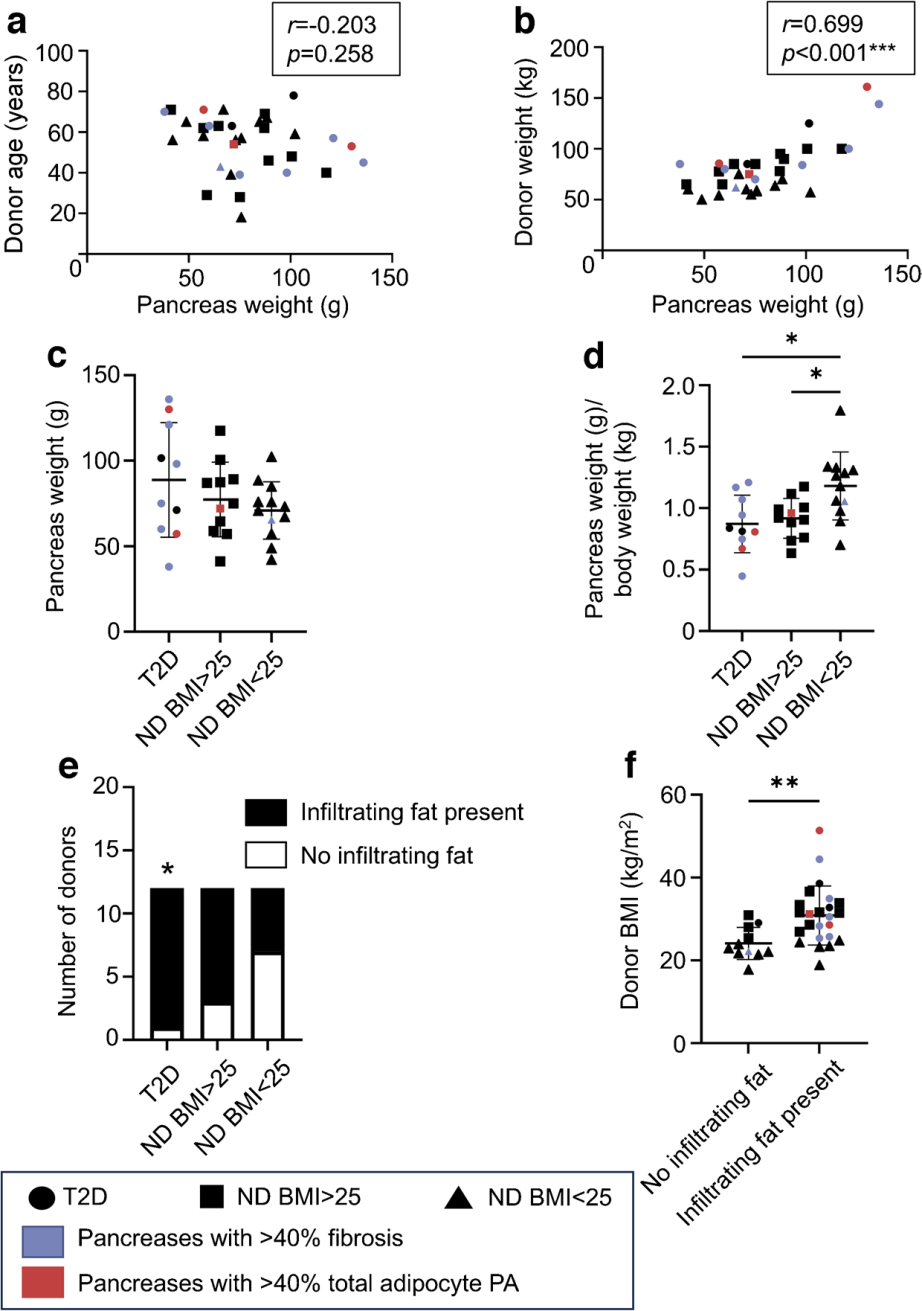
Donor demographics and macroscopic pancreas assessment. Scatter plots indicate that pancreas weight correlated with (**a**) donor age and (**b**) donor weight. (**c**) Pancreas weight and (**d**) pancreas weight normalised to body weight by donor subgroup. (**e**) Number of donors in each subgroup with infiltrating pancreatic fat observed following dissection. (**f**) Donor BMI of pancreases with and without infiltrating fat. Bars indicate mean ± SD. *p<0.05, **p<0.01, ***p<0.001. *n*=33 (**a**–**d**), *n*=35 (**e**, **f**). ND, no history of diabetes; T2D, type 2 diabetes

Pancreas weight following dissection ranged from 38 to 136 g (Fig. 1, ESM Table 1). There was no correlation with age but significant positive correlations with donor weight (Fig. 2a, b), BMI and WC (ESM Fig. 2e, f). Pancreas weights were comparable across all three subgroups (Fig. 2c) but were lower when normalised to body weight in those with BMI >25 kg/m^2^ with and without diabetes compared with those with BMI <25 kg/m^2^ (Fig. 2d).

All pancreases had surrounding fat prior to dissection. Fat infiltration was seen in 24 (69%) organs following dissection from extra-pancreatic fat, being present in ten (91%) organs from overweight donors with diabetes compared with nine (75%) pancreases from overweight donors without diabetes and five (42%) from normal-weight non-diabetic donors (*p*=0.027 vs type 2 diabetes donors) (Fig. 2e). Donors with infiltrating fat were not significantly different in age but had higher BMI and WC (Fig. 2f, ESM Fig. 2g, h).

### Assessment of pancreas adipocyte area

**Fig. 3:**
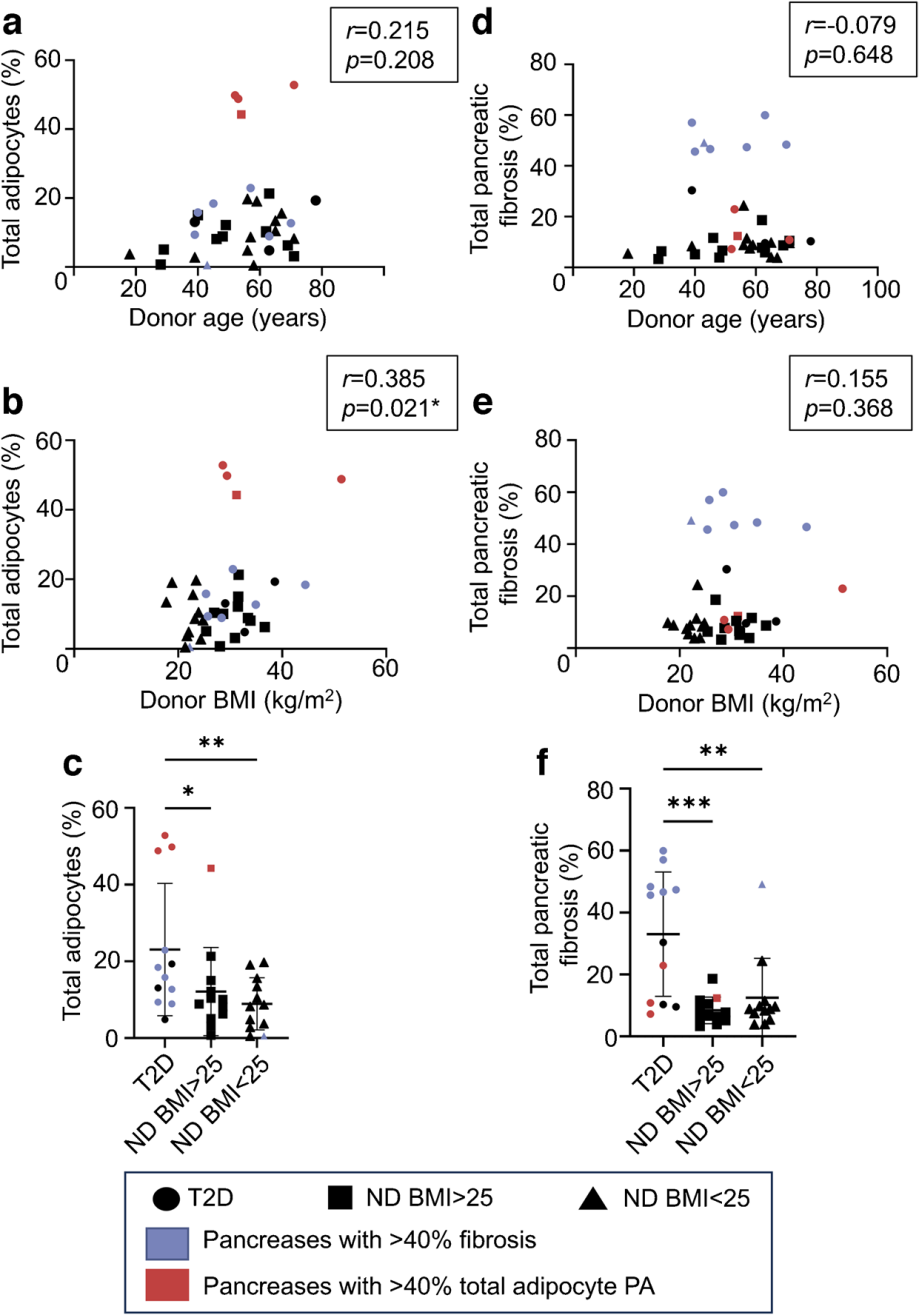
Higher adipocyte and collagen PAs are associated with type 2 diabetes. Total adipocytes plotted against (**a**) donor age and (**b**) donor BMI. (**c**) Total adipocytes by donor subgroup. Total pancreatic collagen PA plotted against (**d**) donor age and (**e**) donor BMI. (**f**) Total collagen by donor subgroup. Bars indicate mean ± SD. *p<0.05, **p<0.01, ***p<0.001. *n*=36. ND, no history of diabetes; T2D, type 2 diabetes

In the overall cohort, adipocyte area was correlated with BMI (*r*=0.385, *p*=0.021) and WC, but not donor age (Fig. 3a, b, ESM Fig. 3a). Adipocyte PA was significantly higher in donors with type 2 diabetes compared with both subgroups without diabetes (Fig. 3c). These associations between adipocyte PA and donor characteristics were comparable when considering extralobular and intralobular fat individually (ESM Fig. 4). Four donors (three with known diabetes and one overweight donor without diabetes, with BMI 31.2 kg/m^2^ and HbA_1c_ 35.5 mmol/mol [5.4%]) had a strikingly high proportion of total adipocytes (>40% of total tissue area), appearing to form a separate cluster (Fig. 3). These were designated ‘high-fat’ pancreases.

### Assessment of collagen deposition

There were no associations between pancreatic fibrosis (quantified by percentage SRFG positivity) and donor age, BMI or WC (Fig. 3d, e, ESM Fig. 3b) within the overall cohort. Pancreatic fibrosis was comparable in donors without diabetes with normal or increased weight, but all quantitative fibrosis measures were significantly higher in donors with type 2 diabetes (Fig. 3f). These associations between pancreatic fibrosis and donor characteristics were comparable when considering acinar, ductal/vascular, peri-islet and intra-islet SRFG PA individually (ESM Fig. 5). A subset of seven pancreases exhibited markedly high SRFG PA (>40% of total tissue area), appearing to form a separate cluster (Fig. 3). These were designated ‘high-fibrosis’ pancreases.

### Islet morphometry analyses

**Fig. 4:**
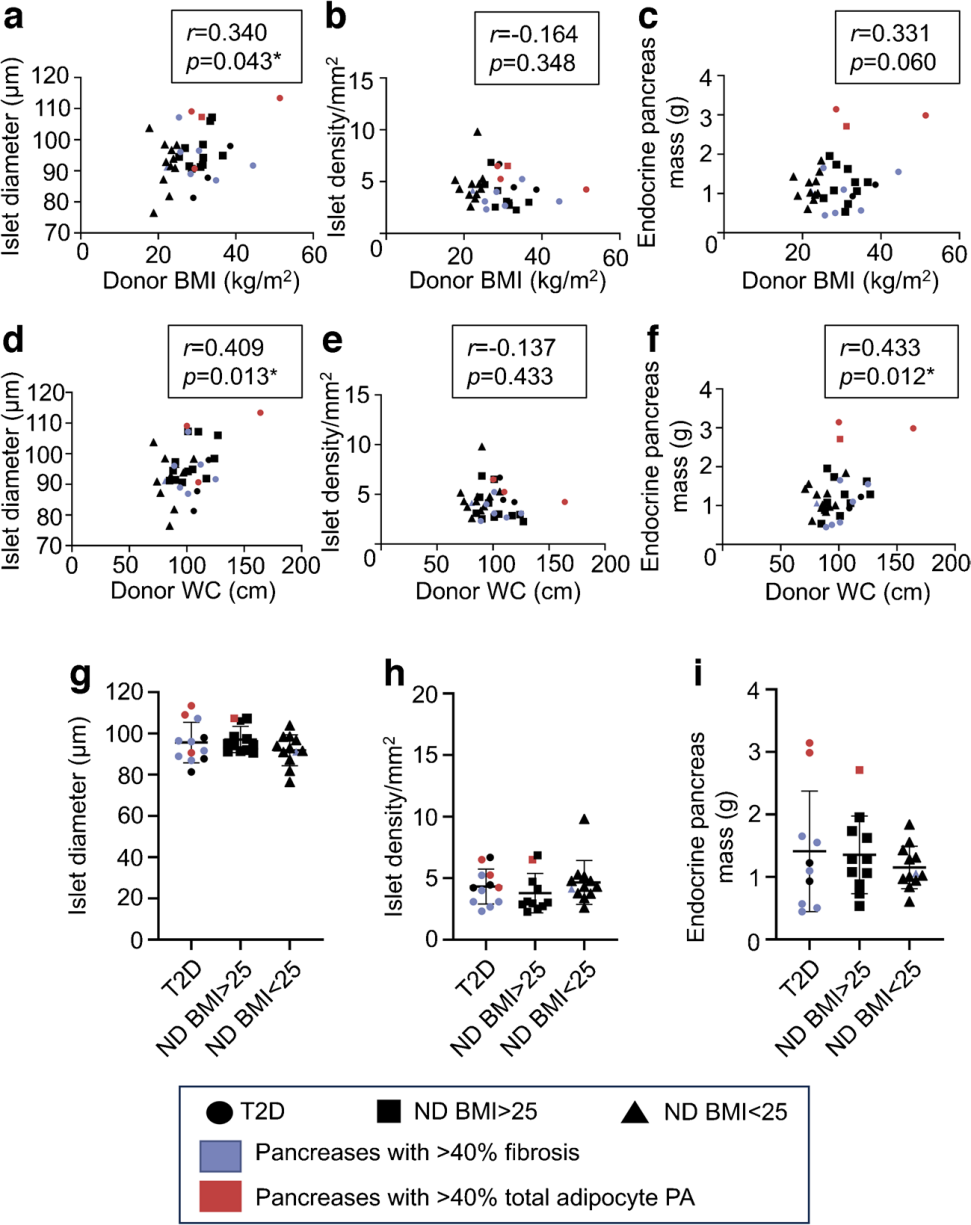
Islet morphometry in the donor cohort. Scatter plots show islet diameter (**a**, **d**), islet density (**b**, **e**) and endocrine pancreas mass (**c**, **f**) plotted against donor BMI (**a**, **b**, **c**) and donor WC (**d**, **e**, **f**). Islet diameter (**g**), islet density (**h**) and endocrine pancreas mass (**i**) by donor subgroup. Bars indicate mean ± SD. *p<0.05. *n*=35 (**a**, **d**, **g**), *n*=36 (**b**, **e**, **h**), *n*=33 (**c**, **f**, **i**). ND, no history of diabetes; T2D, type 2 diabetes

Quantitative analysis of islets in all 16 regions was performed following CGA staining. Islets were defined as CGA-positive structures with an area of >1000 µm^2^. Sampling of all pancreatic regions enabled meaningful estimation of total endocrine mass. There was no correlation between donor age and islet diameter or density or endocrine mass (ESM Fig. 6a–c). Increasing donor BMI and WC were associated with increasing islet diameter and estimated total endocrine mass (Fig. 4a–f). Islet diameter and density and endocrine mass were comparable in those with/without diabetes and/or BMI >25 kg/m^2^ (Fig. 4g–i).

**Fig. 5:**
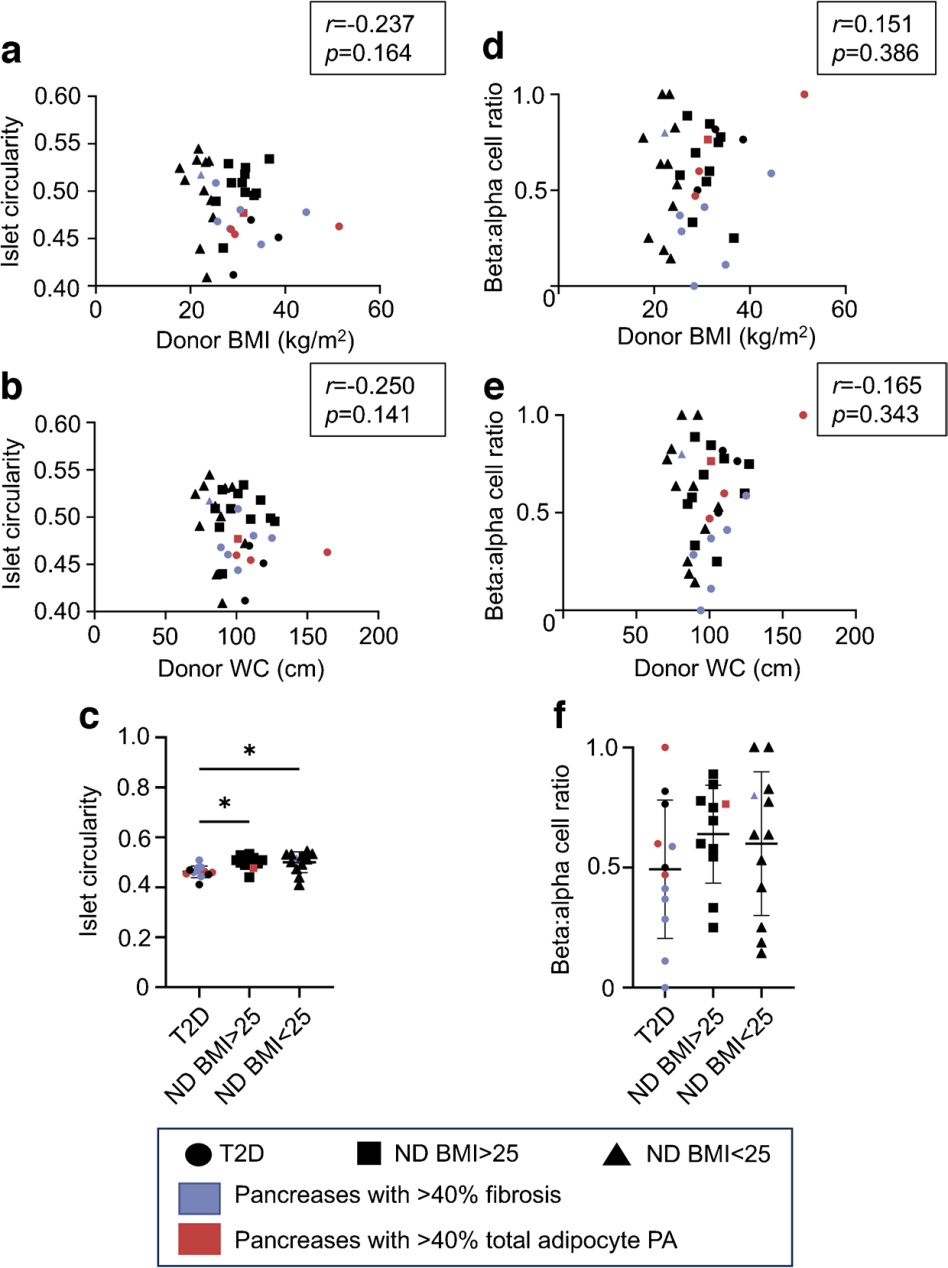
Islet circularity and beta:alpha cell ratio in the donor cohort. Islet circularity plotted against (**a**) donor BMI and (**b**) donor WC. (**c**) Islet circularity by donor subgroup. Beta:alpha cell ratio plotted against (**d**) donor BMI and (**e**) donor WC. (**f**) Beta:alpha cell ratio by donor subgroup. Bars indicate mean ± SD. *p<0.05. *n*=36 (**a**–**c**), *n*=35 (**d**–**f**). ND, no history of diabetes; T2D, type 2 diabetes

Islet circularity was not associated with donor age, BMI or WC but was significantly decreased in donors with diabetes (ESM Fig. 6d, Fig. 5a–c). Endocrine cell types were identified by TEM morphology with estimation of beta:alpha cell ratios. The beta:alpha cell ratio was not associated with donor age, BMI or WC (ESM Fig. 6e, Fig. 5d, e). The beta:alpha cell ratio was numerically lowest in donors with diabetes but was not significantly different from the ratio in those without diabetes (*p*=0.41) (Fig. 5f).

### Intracellular lipid assessment

**Fig. 6:**
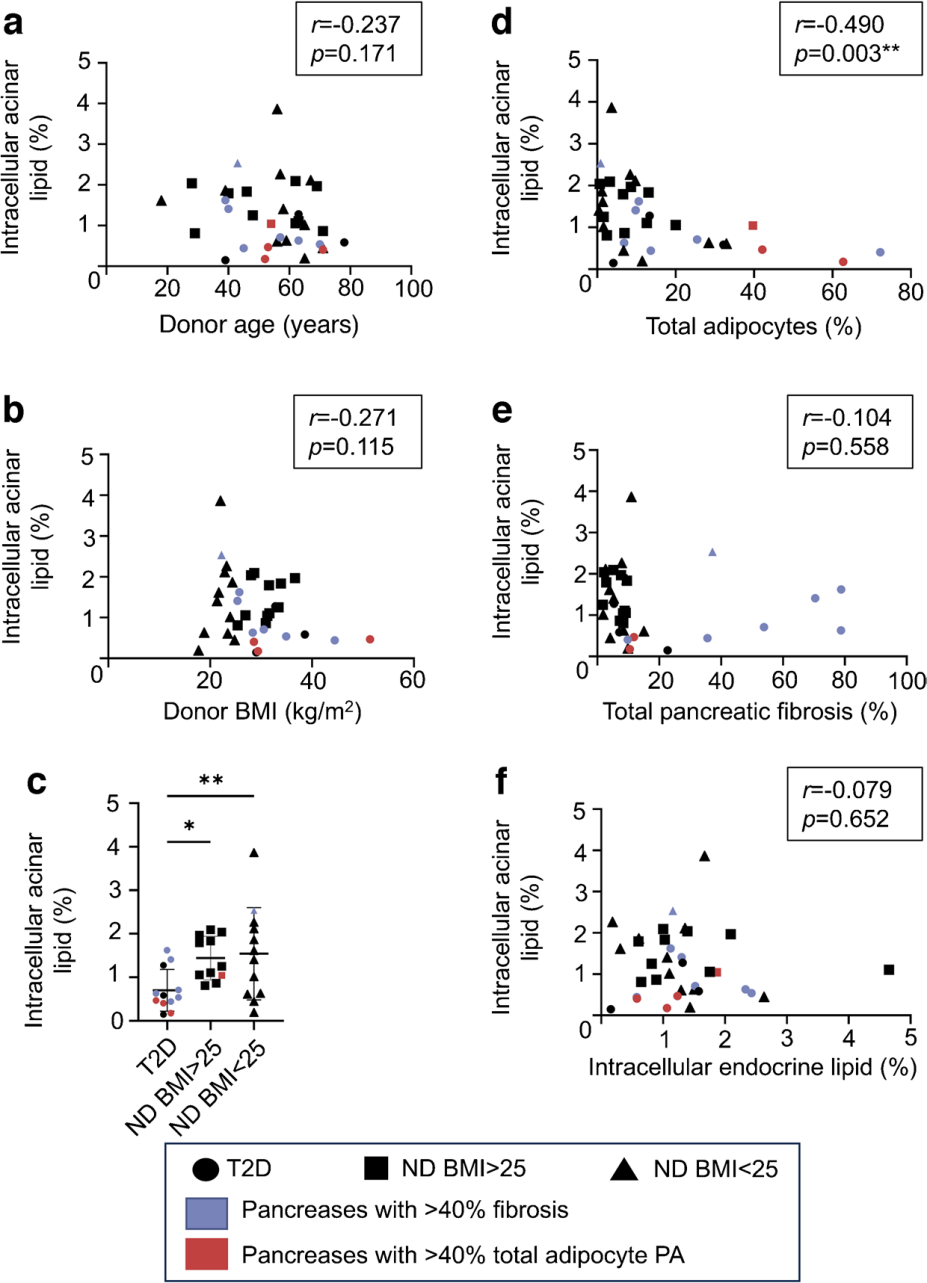
Acinar cell LD PA. Intracellular lipid is expressed as the mean PA of each acinar cell comprising LDs. Scatter plots show the correlation of intracellular acinar lipid PA with (**a**) donor age, (**b**) donor BMI, (**d**) total adipocytes, (**e**) total pancreatic fibrosis and (**f**) intracellular endocrine lipid. (**c**) Acinar LDs stratified by donor subgroup. Correlations with adipocyte and fibrosis PA determined using P4A pancreas region only. Bars indicate mean ± SD. *p<0.05, **p<0.01. *n*=35. ND, no history of diabetes; T2D, type 2 diabetes

In the overall cohort, there was no correlation between mean percentage acinar cell lipid content quantified using TEM and donor age, BMI (Fig. 6a, b) or WC (ESM Fig. 3c). Donors with type 2 diabetes had significantly lower levels of acinar intracellular lipid than donors without diabetes (Fig. 6c). This was in direct contrast to pancreatic adipocyte infiltration with inverse correlations seen between adipocyte area and intra-acinar LD content (*r*=−0.490, *p*=0.003) (Fig. 6d). No correlations between pancreatic fibrosis and acinar cell LD accumulation were observed (Fig. 6e).

**Fig. 7:**
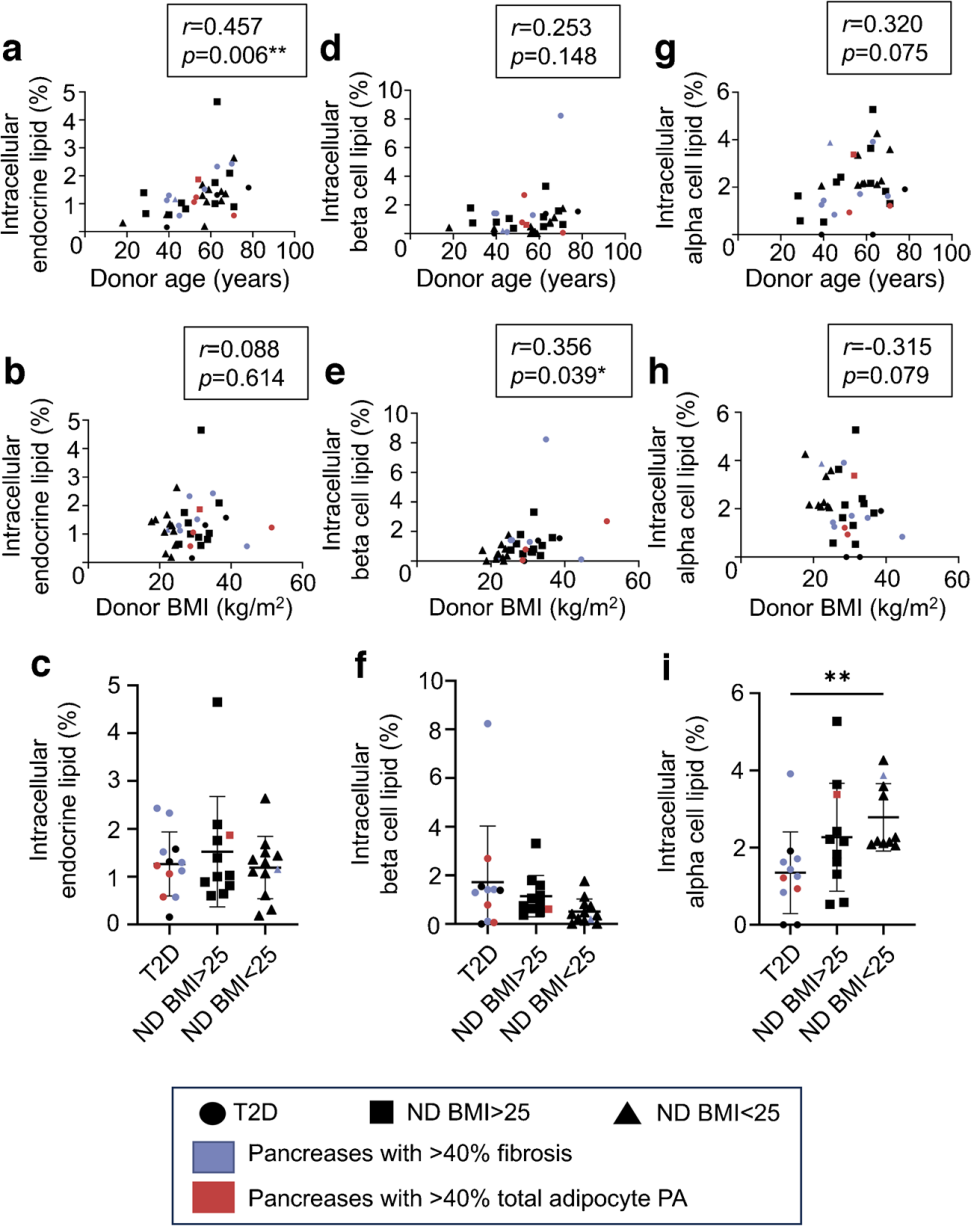
Endocrine cell LD PA. Intracellular lipid is expressed as the mean percentage PA of endocrine cells comprising LDs. Scatter plots show correlation of donor age with (**a**) overall endocrine lipid PA, (**d**) beta cell lipid PA and (**g**) alpha cell lipid PA; and correlation of donor BMI with (**b**) overall endocrine lipid PA, (**e**) beta cell lipid PA and (**h**) alpha cell lipid PA. (**c**, **f**, **i**) Overall endocrine, beta cell and alpha cell lipid PA stratified by donor subgroup. Bars indicate mean ± SD. *p<0.05, **p<0.01. *n*=35 (**a**, **b**, **c**), *n*=34 (**d**, **e**, **f**), *n*=32 (**g**, **h**, **i**). ND, no history of diabetes; T2D, type 2 diabetes

There was a significant correlation between endocrine cell lipid content and donor age but not BMI in the overall cohort (Fig. 7a, b). There were no differences in overall endocrine cell lipid content between normal-weight donors, overweight donors or those with diabetes (Fig. 7c). Endocrine cell lipid content was not associated with acinar intracellular lipid content (Fig. 6f), exocrine adipocyte PA or pancreatic fibrosis (ESM Fig. 7a, b).

To further investigate endocrine cell lipid content, individual endocrine phenotypes were classified by ultrastructural appearance (ESM Fig. 1d, e) and analyses performed by cell type. A possible correlation between increasing beta cell lipid content and BMI but not donor age was seen (Fig. 7d, e). Beta cell lipid content was not significantly higher in donors with type 2 diabetes (Fig. 7f).

There was no correlation between alpha cell lipid content and donor age or BMI (Fig. 7g, h). Donors with diabetes had significantly lower alpha cell lipid content than normal-weight donors without diabetes (Fig. 7i).

No correlations were seen between beta/alpha cell lipid content and exocrine adipocyte areas or pancreatic fibrosis (ESM Fig. 7). Endocrine cell lipid content was not associated with islet morphometry or beta:alpha cell ratio (ESM Fig. 8).

### Comparative analysis of ‘high-fat’ and ‘high-fibrosis’ pancreases

**Fig. 8:**
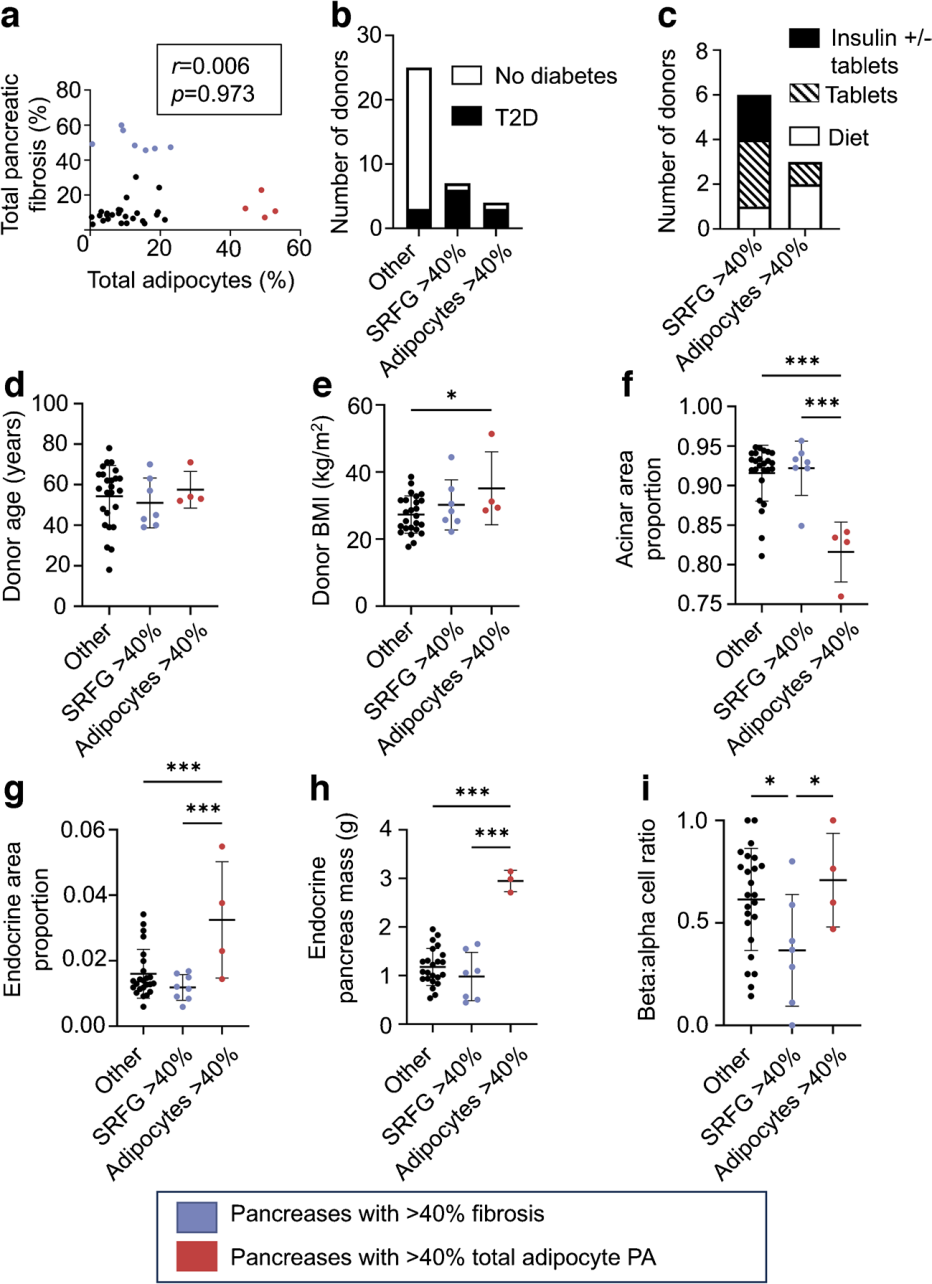
Donor and pancreas parameters stratified by high-fat/high-fibrosis phenotype. (**a**) Scatter plot showing correlation of total adipocyte PA with total pancreatic collagen PA. (**b**) Donor diabetes status, (**c**) glucose-lowering medication, (**d**) donor age, (**e**) donor BMI, (**f**) acinar area proportion, (**g**) endocrine area proportion, (**h**) endocrine pancreas mass and (**i**) beta:alpha cell ratio in high-fat/high-fibrosis/other pancreases. Bars indicate mean ± SD. *p<0.05, ***p<0.001. *n*=9 (**c**), *n*=33 (**h**), *n*=35 (**i**), *n*=36 (all other figure parts). T2D, type 2 diabetes

In the overall cohort SRFG and adipocyte PA were not correlated (Fig. 8a). High-fibrosis and high-fat organs appeared to constitute distinct clusters, with no pancreases with >40% fibrosis also having >40% adipocyte PA (Fig. 8a). This was supported by *k*-means cluster analysis which divided pancreases into those with fibrosis/fat PA greater than or less than 40%. To examine whether high-fat and high-fibrosis donors may represent differing phenotypes, analyses were repeated with stratification by pancreas subtype rather than donor BMI or diabetes status.

Seventy-five per cent of ‘high-fat’ and 86% of ‘high-fibrosis’ pancreases were from donors with diabetes vs 12% from non-high-fat/non-high-fibrosis donors (Fig. 8b). All ‘high-fibrosis’ donors (where diagnosis date was known) had a longer duration of diabetes than ‘high-fat’ donors (ESM Fig. 9a), and both individuals with insulin-treated diabetes in the overall cohort had highly fibrotic pancreases (Fig. 8c).

While there was no difference in donor age between the three subgroups, ‘high-fat’ donors had higher BMI and WC (Fig. 8d, e, ESM Fig. 9b). In the overall cohort, increasing adipocyte PA was associated with increased endocrine and reduced acinar area (*r*=−0.762, *p*<0.001) (ESM Fig. 10a, b). Higher adipocyte PA was associated with increased islet density, diameter and endocrine mass (*r*=0.749, *p*<0.001) (ESM Fig. 10c–e). ‘High-fat’ donors had reduced acinar PA with increased islet density and diameter, associated with increased endocrine PA and estimated endocrine mass (Fig. 8f–h, ESM Fig. 9c, d). There appeared to be no impact of ‘high fibrosis’ on endocrine mass (Fig. 8h).

Reducing beta:alpha cell ratio was seen with increasing total pancreatic fibrosis in the overall cohort (and specifically in the ‘high-fibrosis’ donors [*r*=−0.88*, p*=0.02]) whereas no relationship with pancreatic adipocytosis was seen (ESM Fig. 11). Ratio was significantly reduced in ‘high-fibrosis’ but not ‘high-fat’ donors (Fig. 8i).

## Discussion

Through quantitative histological and electron microscopy analysis of a cohort of optimally retrieved and systematically biopsied deceased organ donors we have characterised pancreatic fat and fibrosis in overweight individuals with and without type 2 diabetes in comparison with control donors with BMI <25 kg/m^2^ and normal HbA_1c_. Pancreatic adipocytosis increased with donor adiposity (BMI and WC) and was associated with type 2 diabetes. Pancreatic fibrosis was also associated with type 2 diabetes, but not with BMI, WC or pancreas fat. Seventy-five per cent of donors with diabetes had >40% fat or >40% fibrosis PA but none had both, suggesting that these may represent two separate type 2 diabetes phenotypes. Intra-acinar cell LD area fell incrementally with increasing adipocyte PA, being lowest in type 2 diabetes. Endocrine LD area was not significantly increased in type 2 diabetes.

Increasing pancreatic adipocytosis with increasing BMI has been consistently reported in previous histological studies and in a large clinical cohort using computed tomography (CT) scanning [26, 27]. These studies concluded that adipocyte PA is not increased further in type 2 diabetes. Image analysis combining 16 anatomically defined regions throughout the whole pancreas in all donors has not been undertaken previously, with analyses restricted to head, body and/or tail [13, 26]. In the current analysis, extralobular and intralobular adipocyte PA were quantified, with both being significantly higher in type 2 diabetes vs donors without diabetes. Our analysis also included macroscopic pancreas assessment of infiltrating fat which was seen most commonly in donors with diabetes. We propose that the irregular pancreatic outline on MRI reported by Al-Mrabeh et al in type 2 diabetes may represent interdigitating fat, infiltrating between pancreatic lobes [7].

In this adult cohort pancreas weight was not associated with donor age but increased with body weight, BMI and WC. Unadjusted pancreas weight was comparable in the presence and absence of type 2 diabetes, but when corrected for total body weight was lower in high BMI donors regardless of diabetes status. In a previous CT imaging study, pancreas volume increased until age 20 years and then remained constant until age 60 years [27]. Increasing pancreatic volume with increasing BMI was described in the clinical study, mirroring pancreatic weights following careful dissection from surrounding fat in the current study. Volume calculated from CT imaging and in an MRI study was significantly lower in participants with type 2 diabetes compared with BMI-matched control participants without diabetes [6, 27], although in a relatively small study including 29 individuals with type 2 diabetes, CT-determined pancreatic volume with or without correction for body surface area was not reduced in contrast to significantly smaller pancreases in type 1 diabetes [28]. In keeping with our own findings, a previous histological study reported no change in pancreas weight in type 2 diabetes vs control donors [13]. The discrepancy between clinical imaging and histopathology studies suggests that interdigitating fat may be pathognomonic of type 2 diabetes, included within pancreas weight but potentially excluded from pancreatic volume estimations [6], although relatively lower sample sizes in histological studies necessitate further work to confirm or refute this.

Pancreatic fibrosis was associated with type 2 diabetes but not donor BMI or WC. Increased intralobular fibrosis has previously been reported in type 2 diabetes [13] but, in this quantitative analysis, increased fibrosis was present in all pancreatic compartments including peri-/intra-islet regions.

Islet diameter increased with increasing BMI/WC and estimated endocrine mass was positively correlated with WC in the overall cohort. Increasing pancreatic adipocytosis and ‘high-fat’ phenotype were associated with reduced acinar proportion and increased islet diameter and density and estimated endocrine mass. This suggests an adaptive expansion of islet mass in the pancreatic endocrine compartment in response to insulin resistance, in parallel with adipocyte replacement of acinar volume. It may be that it is the loss of acinar cell mass that results in the apparent total pancreas volume reduction in type 2 diabetes imaging studies despite maintained overall pancreatic weight postmortem*.* No loss of acinar mass or endocrine compartment expansion was seen with increasing pancreatic fibrosis or in ‘high-fibrosis’ organs.

Type 2 diabetes was not associated with reduction in islet diameter or density or estimated endocrine mass but was associated with reduced islet circularity. Decreased overall islet volume in type 2 diabetes has previously been reported along with disrupted islet morphology, particularly in the presence of intra-islet islet amyloid polypeptide deposits [29], but quantification of islet size and circularity systematically combining 16 anatomical pancreatic regions has not previously been undertaken. Although our data support relative maintenance of overall endocrine mass in type 2 diabetes, with a potentially greater impact of disrupted morphology associated with pancreatic adipocyte infiltration/fibrosis, it is accepted that there is a decrease in beta cell mass [30]. Both ‘high-fat’ and ‘high-fibrosis’ phenotypes were associated with decreased islet circularity. Beta:alpha cell ratio was significantly reduced in ‘high-fibrosis’ donors and all individuals with insulin-treated diabetes were in this subgroup.

While it may be that the natural history of type 2 diabetes involves both adipocytosis and fibrosis, we propose that high-fibrosis and high-fat organs in donors with antemortem diagnosis of type 2 diabetes represent separate phenotypes. Different subtypes of type 2 diabetes have been proposed, including an insulin resistance phenotype with less severe beta cell dysfunction; and a relatively low BMI/insulin sensitive subtype with more marked beta cell insufficiency and earlier progression to exogenous insulin therapy [31, 32]. Type 3c diabetes secondary to chronic pancreatitis and associated with pancreatic fibrosis is often mis-classified as type 2 diabetes [33]. The current cohort did not, however, have history of chronic pancreatitis or organ pathology suggestive of this underlying diagnosis. Although one donor had a single episode of acute pancreatitis 9 years prior to death, there was no pathological evidence of chronic pancreatitis and the donor was not in the ‘high-fibrosis’ group. Indeed, in a systematic review including 50 original studies it was concluded that type 2 diabetes is associated with diabetic exocrine pancreatopathy comprising fibrosis without loss of pancreatic volume or any symptomatic/pathological features of chronic pancreatitis [34].

Using combined histological and ultrastructural analyses, we have shown for the first time an inverse association between pancreatic adipocyte proportion and LD PA within acinar cells. The presence of lipid droplets within acinar cells has been reported in mice fed a high-fat diet compared with control mice, in parallel with increased adipocyte PA [35]. In the same study adipocyte area was shown to correlate with overall pancreatic triglyceride content in human pancreases. The authors concluded that excess pancreatic fat is largely stored within adipocytes and that secreted products from these cells, including adipokines and leptin, may contribute to islet dysfunction in type 2 diabetes. Although the mechanism underlying decreasing intra-acinar cell lipid content with increasing adipocyte accumulation remains obscure, acinar cell lipid content is low in type 2 diabetes [10] and intra-acinar LD accumulation cannot therefore be implicated in diabetes development.

Overall, endocrine cell lipid content increased with age in keeping with a previous report in human tissue [10], but was not higher in donors with diabetes. In the previous study [10], a relative increase in islet vs exocrine cell lipid content was reported in type 2 diabetes but this was largely attributable to lower LD numbers in acinar cells, with the study data aligning with our own. We have for the first time attempted to interrogate lipid content in alpha and beta cells separately in fixed human tissue using TEM ultrastructural phenotyping. Beta cell lipid content was associated with BMI in agreement with a study in isolated human islets [36] but was not significantly higher in donors with diabetes. Interestingly, alpha cell lipid content was significantly lower in donors with type 2 diabetes. We conclude that, although there may be some increase in beta cell lipid content with increasing BMI, intra-endocrine cell LDs do not appear to be a primary driver of progression to type 2 diabetes.

Various interacting factors have been reported to influence pancreatic fat, including ageing, inflammation, metabolic heterogeneity, MASLD, diet, alcohol and smoking [37–40], and may also affect pancreatic fibrosis development, although this is less well understood [16, 41]. Genetic factors may influence sensitivity of beta cells to fatty acids and chemokines secreted by adipocytes, since not all individuals with pancreatic adipocytosis develop type 2 diabetes [39]. Expanded studies in larger cohorts, combining deep spatial phenotyping with comprehensive donor clinical history and genetic data, will be necessary to further characterise these proposed ‘high-fat’ and ‘high-fibrosis’ type 2 diabetes phenotypes and to elucidate the mechanisms leading to endocrine dysfunction.

Detailed quantitative histological analysis of samples from anatomically defined regions throughout the pancreas and parallel subcellular TEM analysis are study strengths, although future investigation of regional differences in exocrine and endocrine pathology will be important. Limitations include the small cohort size, reducing the study power and necessitating caution in interpreting the significance of findings, particularly regarding the high-fat and high-fibrosis subgroups. The donor sex ratio was also skewed, with the majority of type 2 diabetes donors being male and the majority without diabetes being female. In addition, our cohort was limited to white donors precluding generalisability to other ethnicities. Higher pancreatic fat content in male individuals has been reported, although this difference between sexes was not present in type 2 diabetes [42]. It is important to note that the clinical implications of pathological findings including differences in islet mass, circularity and beta:alpha cell ratios cannot be assumed without further data on endocrine function.

In conclusion, systematic whole-organ analysis of pancreatic adipocyte PA, fibrosis, islet morphometry and intracellular lipid content has confirmed an association between elevated BMI and increased pancreatic adipocytosis together with higher endocrine mass in parallel with decreased acinar cell lipid content and increased beta cell lipid content. Type 2 diabetes was associated with elevated pancreatic fat and fibrosis with maintained islet mass. High-fibrosis and high-fat organs appeared to constitute separate phenotypic subtypes. Our findings support the presence of diverse pathogenic pathways in type 2 diabetes development, and the future potential for mechanistically informed personalised disease-modifying therapies targeting each phenotype.

## Supplementary Information

Below is the link to the electronic supplementary material.

ESM (PDF 849 KB)

## Data Availability

The datasets generated during and/or analysed during the current study are available from the corresponding author upon reasonable request.

## Abbreviations

AI: Artificial intelligence
CGA: Chromogranin A
CT: Computed tomography
LD: Lipid droplet
MASLD: Metabolic dysfunction-associated steatotic liver disease
P4A region: P4 anterior region
PA: Proportional area
SRFG: Sirius Red Fast Green
TEM: Transmission electron microscopy
WC: Waist circumference
