# Supplementary material for: Quantitative analysis of human adult pancreatic histology reveals separate fatty and fibrotic phenotypes in type 2 diabetes
Source: Diabetologia. 2025 Sep 24;68(12):2840–53. doi: 10.1007/s00125-025-06547-8 (PMC12594667; doi:10.1007/s00125-025-06547-8)

ESM Table 1. Summary of donor characteristics.

Donor [a]	Unique identifier	Donor type [b]	Age (years)	Sex	Ethnic origin	Height (cm)	Weight (kg)	BMI (kg/m^2)	Waist circumference (cm)	History of diabetes [c]	Glucose lowering medication history	Duration of diabetes (months)	Family history of diabetes [c]	History of pancreatitis	Pancreas weight (g)	Pancreas weight (g): donor weight (kg)
T2D1	PT0267-0079	DBD	39	Male	White	165	70	25.71	89	T2D	Medication	28	No	No	75	1.07
T2D2	PT0267-0043	DCD	39	Male	White	176	90	29.05	106	T2D	Medication	Not reported	Yes, type unknown	Yes, acute	Not recorded	N/A
T2D3	PT0267-0077	DBD	40	Male	White	182	84	25.36	101	T2D	Diet	Not reported	Yes, T1D	No	98.2	1.17
T2D4	PT0267-0075	DBD	45	Male	White	180	144	44.44	125	T2D	Medication	Not reported	Yes, T1D and T2D	No	135.9	0.94
T2D5	PT0267-0045	DCD	52	Male	White	175	90	29.39	110	T2D	Diet	12	No	No	Not recorded	N/A
T2D6	PT0267-0082	DBD	53	Male	White	177	161	51.39	164	T2D	Medication	15	Yes, type unknown	No	130	0.81
T2D7	PT0267-0080	DCD	57	Male	White	181	100	30.52	112	T2D	Medication	102	No	No	121	1.21
T2D8	PT0267-0081	DBD	63	Male	White	168	80	28.34	94	T2D	Insulin +/- medication	312	Yes, T1D and T2D	No	60	0.75
T2D9	PT0267-0026	DBD	63	Female	White	161	85	32.79	109	T2D	Not reported	Not reported	Yes, T2D	No	71.2	0.84
T2D10	PT0267-0020	DBD	70	Female	White	156	85	34.93	101	T2D	Insulin +/- medication	Not reported	Yes, T2D	No	38	0.45
T2D11	PT0267-0051	DBD	71	Male	White	173	85.5	28.57	100	T2D	Diet	1	No	No	57.3	0.67
T2D12	PT0267-0064	DCD	78	Male	White	180	125	38.58	119	T2D	Medication	Not reported	No	No	101.5	0.81
**Summary**		4 (33%) DCD	Mean 55.83 ±13.43	2 (17%) female	12 (100%) white	Mean 172.83 ±8.49	Mean 99.96 ±28.12	Mean 33.26 ±7.93	Mean 110.83 ±19.53		2 (17%) insulin controlled	Mean 78 (range 1-312) months diabetes duration	7 (58%) with family history	1 (8%) with history of pancreatitis	Mean 88.81 ±33.51	Mean 0.87 ±0.23
BMIH1	PT0267-0024	DBD	28	Female	White	178	85	28	90	No	N/A	N/A	No	No	75.1	0.88
BMIH2	PT0267-0055	DBD	29	Female	White	160	65	25.39	88	No	N/A	N/A	No	No	58.9	0.91
BMIH3	PT0267-0025	DBD	40	Female	Unknown	178	100	31.56	124	No	N/A	N/A	No	No	117.6	1.18
BMIH4	PT0267-0008	DBD	46	Female	White	163	90	33.87	110	No	N/A	N/A	No	No	89.2	0.99
BMIH5	PT0267-0019	DBD	48	Female	White	173	100	33.41	127	No	N/A	N/A	No	No	100.6	1.01
BMIH6	PT0267-0023	DBD	49	Male	Unknown	172	93.1	31.47	117	No	N/A	N/A	No	No	Not recorded	N/A
BMIH7	PT0267-0012	DCD	54	Female	White	155	75	31.22	101	No	N/A	N/A	No	No	72.1	0.96

BMIH8	PT0267-0041	DCD	62	Male	White	170	77.8	26.92	90	No	N/A	N/A	Yes, T2D	No	57.2	0.74
BMIH9	PT0267-0030	DBD	62	Male	White	165	78	28.65	96	No	N/A	N/A	No	No	87.1	1.12
BMIH10	PT0267-0021	DBD	63	Female	White	164	85	31.6	101	No	N/A	N/A	No	No	64.6	0.76
BMIH11	PT0267-0066	DBD	69	Female	White	161	95	36.65	105	No	N/A	N/A	Yes, T2D	No	87.4	0.92
BMIH12	PT0267-0034	DBD	71	Female	White	145	65	30.92	85	No	N/A	N/A	Yes, T2D	No	41.2	0.63
**Summary**		2 (17%) DCD	Mean 51.75 ±14.41	9 (75%) female	10 (100%) white	Mean 165.33 ±9.63	Mean 84.08 ±12.18	Mean 30.81 ±3.15	Mean 102.83 ±14.17				3 (25%) with family history	0 with history of pancreatitis	Mean 77.36 ±21.78	Mean 0.92 ±0.16
BMIL1	PT0267-0018	DBD	18	Female	White	165	59	21.67	81	No	N/A	N/A	No	No	75.8	1.28
BMIL2	PT0267-0001	DBD	39	Female	White	157	60	24.34	74	No	N/A	N/A	Yes, T2D	No	70.7	1.18
BMIL3	PT0267-0074	DCD	43	Female	White	167	62	22.23	81	No	N/A	N/A	No	No	65.5	1.06
BMIL4	PT0267-0016	DBD	56	Female	White	158	55	22.03	86	No	N/A	N/A	No	No	73	1.33
BMIL5	PT0267-0062	DBD	56	Female	White	160	60	23.44	90	No	N/A	N/A	Yes, T2D	No	42	0.70
BMIL6	PT0267-0007	DBD	57	Female	White	158	58	23.23	92	No	N/A	N/A	No	No	75.8	1.31
BMIL7	PT0267-0029	DBD	58	Female	White	159	54	21.36	77	No	N/A	N/A	No	No	57.2	1.06
BMIL8	PT0267-0056	DCD	59	Male	White	174	57	18.83	85	No	N/A	N/A	Yes, T2D	No	102.2	1.79
BMIL9	PT0267-0009	DCD	65	Female	White	168	50	17.72	71	No	N/A	N/A	No	No	48.8	0.98
BMIL10	PT0267-0017	DBD	65	Female	White	163	63.5	23.9	97	No	N/A	N/A	No	No	84.8	1.34
BMIL11	PT0267-0003	DBD	67	Male	White	175	70	22.86	89	No	N/A	N/A	No	No	88.4	1.26
BMIL12	PT0267-0040	DBD	71	Female	White	174	75	24.77	106	No	N/A	N/A	No	No	67	0.89
**Summary**		3 (25%) DCD	Mean 54.5 ±14.76	10 (83%) female	12 (100%) white	Mean 164.83 ±6.75	Mean 60.29 ±6.84	Mean 22.20 ±2.12	Mean 85.75 ±9.92				3 (25%) with family history	0 with history of pancreatitis	Mean 70.93 ±16.75	Mean 1.18 ±0.28

[a] BMIH: BMI high (>25 kg/m^2); BMIL: BMI low (<25 kg/m^2). [b] DBD: Donation after brainstem death. DCD: Donation after circulatory death. [c] T1D: type 1 diabetes. T2D: type 2 diabetes. Data are mean ± SD or n(%).

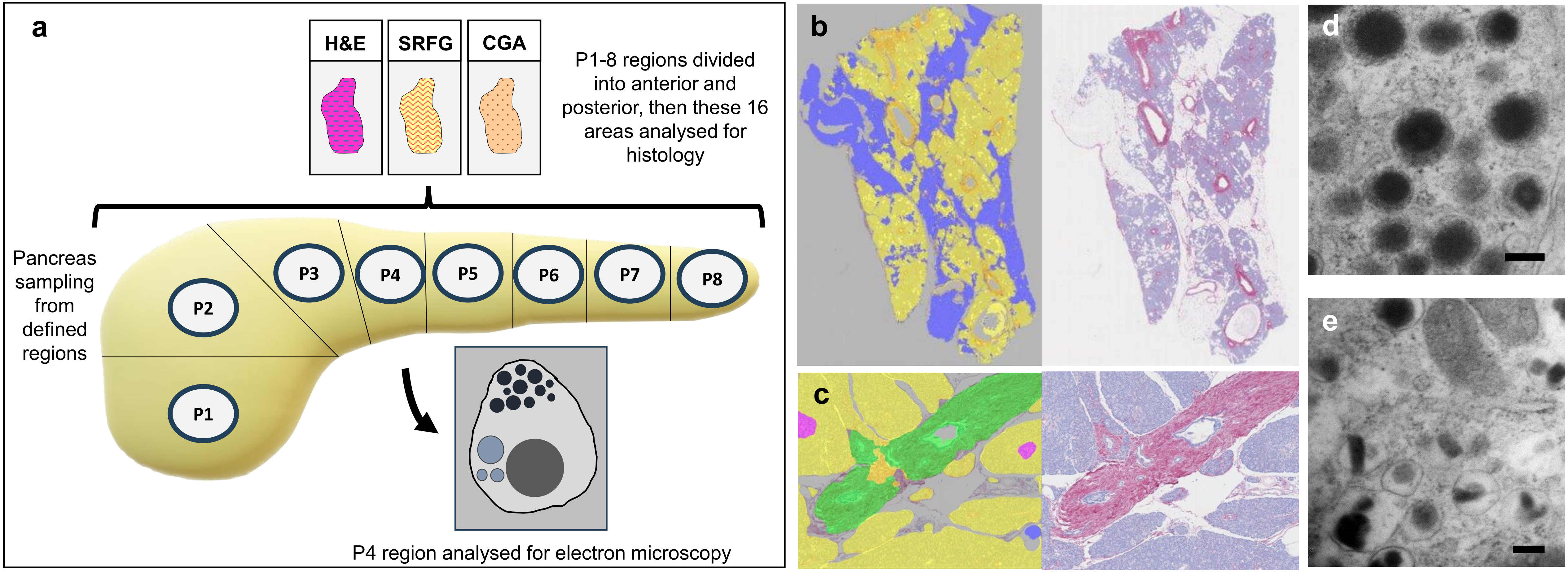

ESM Fig. 1. Pancreas sampling and quantitative histological assessment. (a): Schematic of sampling procedure from the pancreas for downstream analyses. (b-c): Representative images illustrating tissue segmentation of (b) extra-lobular adipocytes (blue), intra-lobular adipocytes (light yellow), (c) collagen (green) and islets (pink) from pancreas parenchyma (dark yellow), via the HALO image analysis platform. (d-e): Representative images of granule morphology in (d) alpha cells and (e) beta cells. Scale bars: 200 nm.

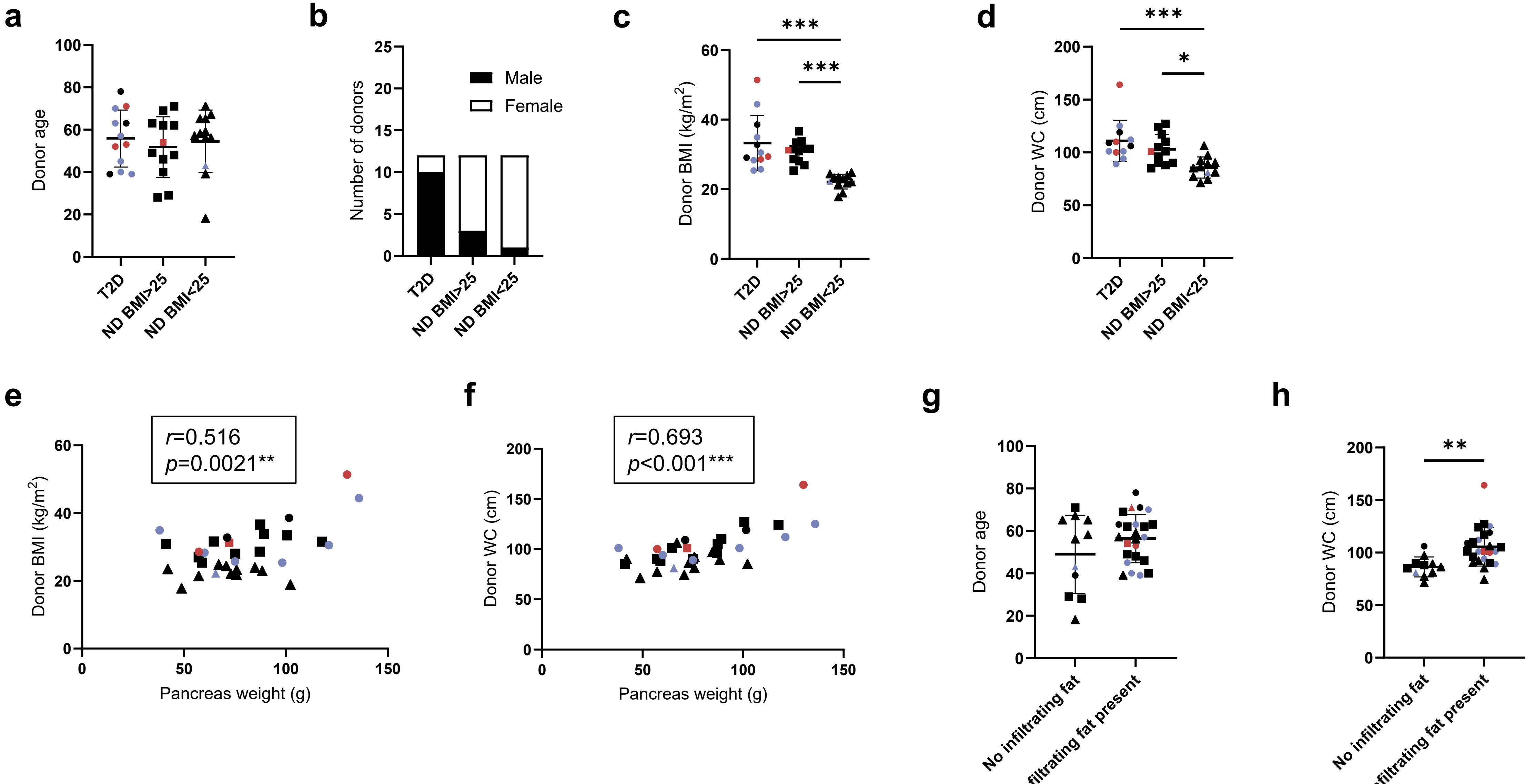

ESM Fig. 2. Donor demographics and macroscopic pancreas assessment.
(a): Donor age, (b): donor sex, (c): donor BMI, (d): donor waist circumference by subgroup. Scatter plots indicate pancreas weight correlated with (e): donor BMI, (f): donor waist circumference. (g): Donor age, (h): donor waist circumference of pancreases with and without infiltrating fat observed following dissection. Bars indicate mean +/-SD. *r*: Pearson's correlation coefficient. **p*<0.05, ***p*<0.01, ****p*<0.001. n=33 (e-f), n=35 (g-h), n=36 (all other figures). ND, no history of diabetes; T2D, type 2 diabetes.

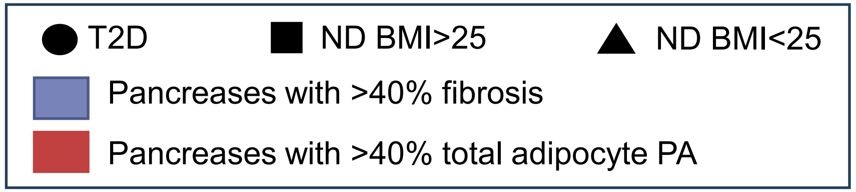

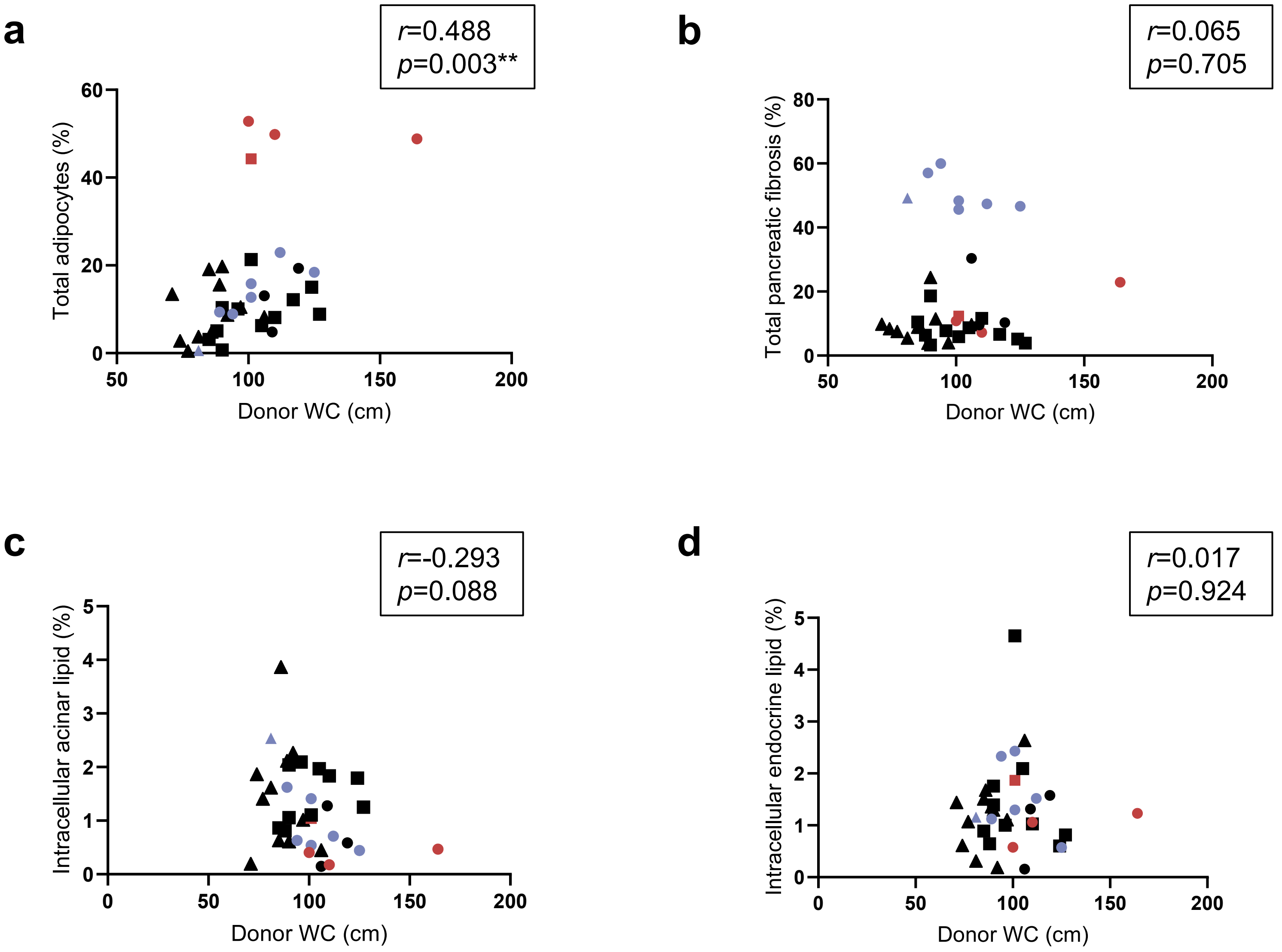

ESM Fig. 3. Donor waist circumference versus adipocyte, fibrosis, and lipid droplet PA. Donor waist circumference is plotted against (a): total adipocyte PA, (b): total pancreatic fibrosis PA, (c): intracellular acinar lipid, and (d): intracellular endocrine lipid. *r*: Pearson's correlation coefficient. ***p*<0.01. n=35 (c-d), n=36 (a-b). ND, no history of diabetes; T2D, type 2 diabetes.

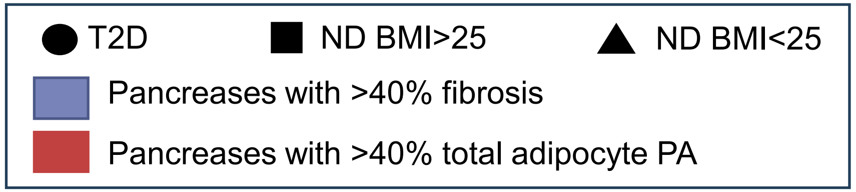

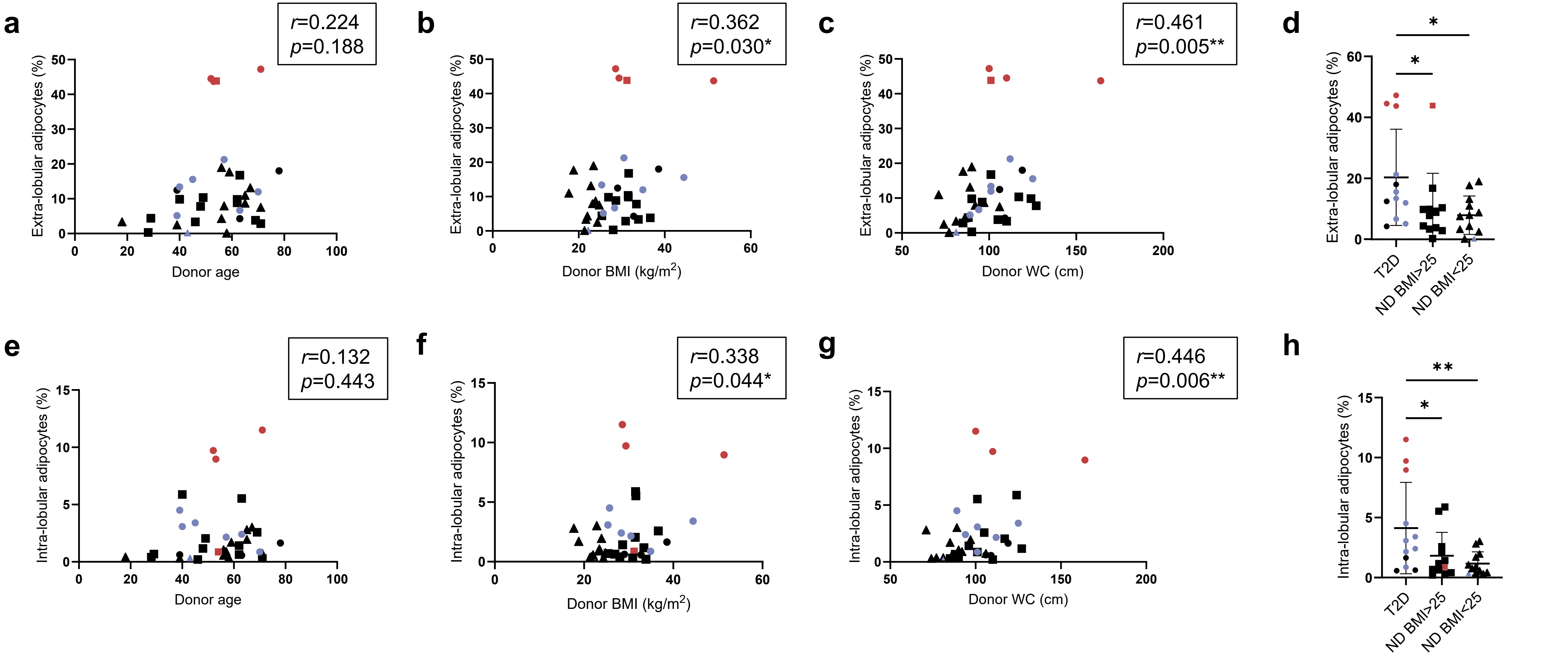

ESM Fig. 4. Associations of extra- and intra-lobular adipocyte proportional areas (PAs) with donor characteristics.

Extra-lobular adipocyte PA plotted against (a): donor age, (b): donor BMI, (c): donor waist circumference. Intra-lobular adipocyte PA plotted against (e): donor age, (f): donor BMI, (g): donor waist circumference. Higher (d): extra-lobular, and (h): intra-lobular adipocyte percentages were present in the T2D subgroup. Bars indicate mean +/-SD. *r*: Pearson's correlation coefficient. *$p<0.05$, **$p<0.01$. n=36. ND, no history of diabetes; T2D, type 2 diabetes.

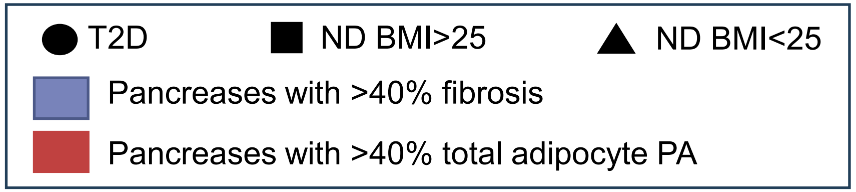

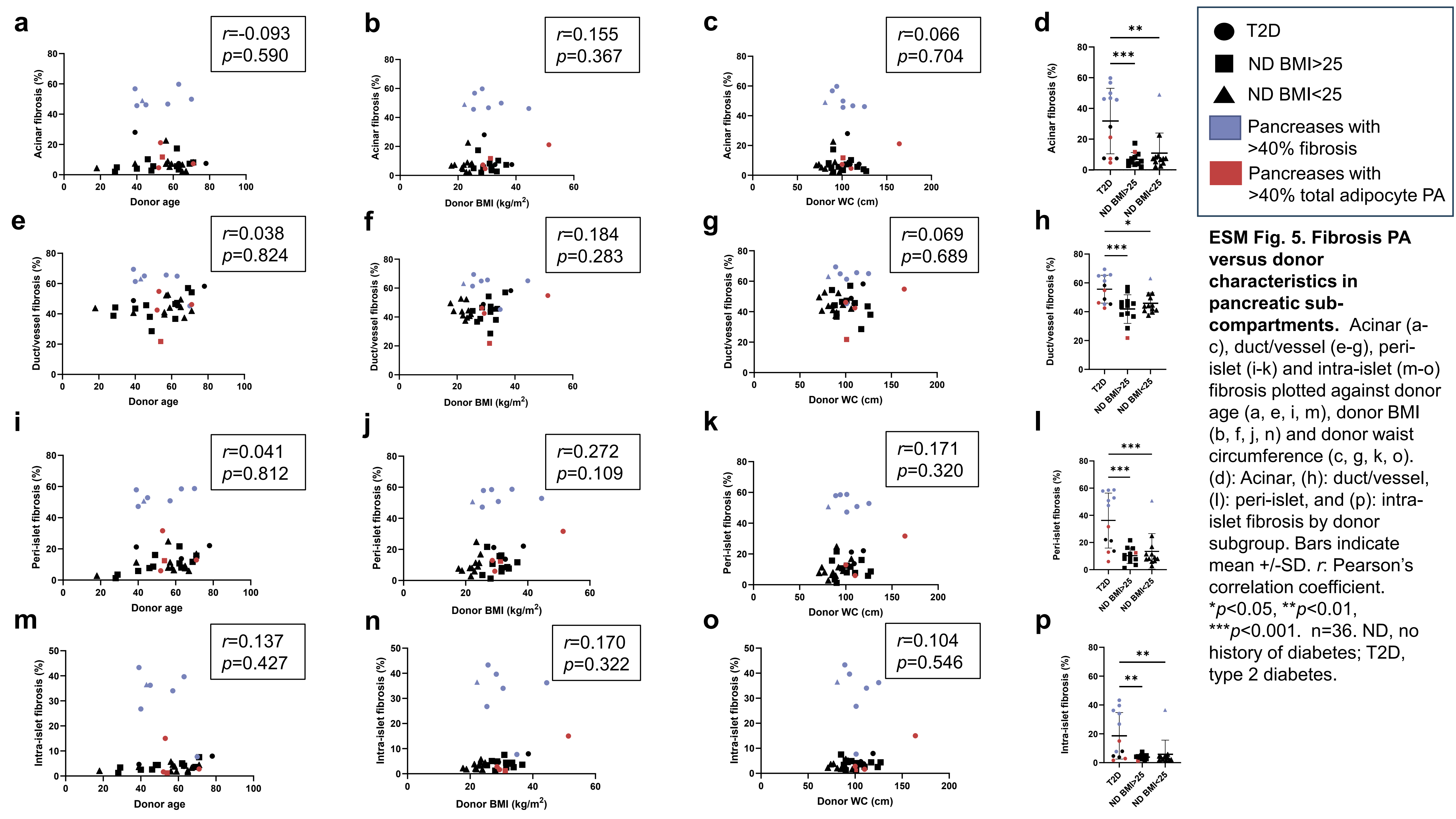

ESM Fig. 5. Fibrosis PA versus donor characteristics in pancreatic sub-compartments. Acinar (a-c), duct/vessel (e-g), peri-islet (i-k) and intra-islet (m-o) fibrosis plotted against donor age (a, e, i, m), donor BMI (b, f, j, n) and donor waist circumference (c, g, k, o). (d): Acinar, (h): duct/vessel, (l): peri-islet, and (p): intra-islet fibrosis by donor subgroup. Bars indicate mean +/-SD. *r*: Pearson's correlation coefficient. **p*<0.05, ***p*<0.01, ****p*<0.001. n=36. ND, no history of diabetes; T2D, type 2 diabetes.

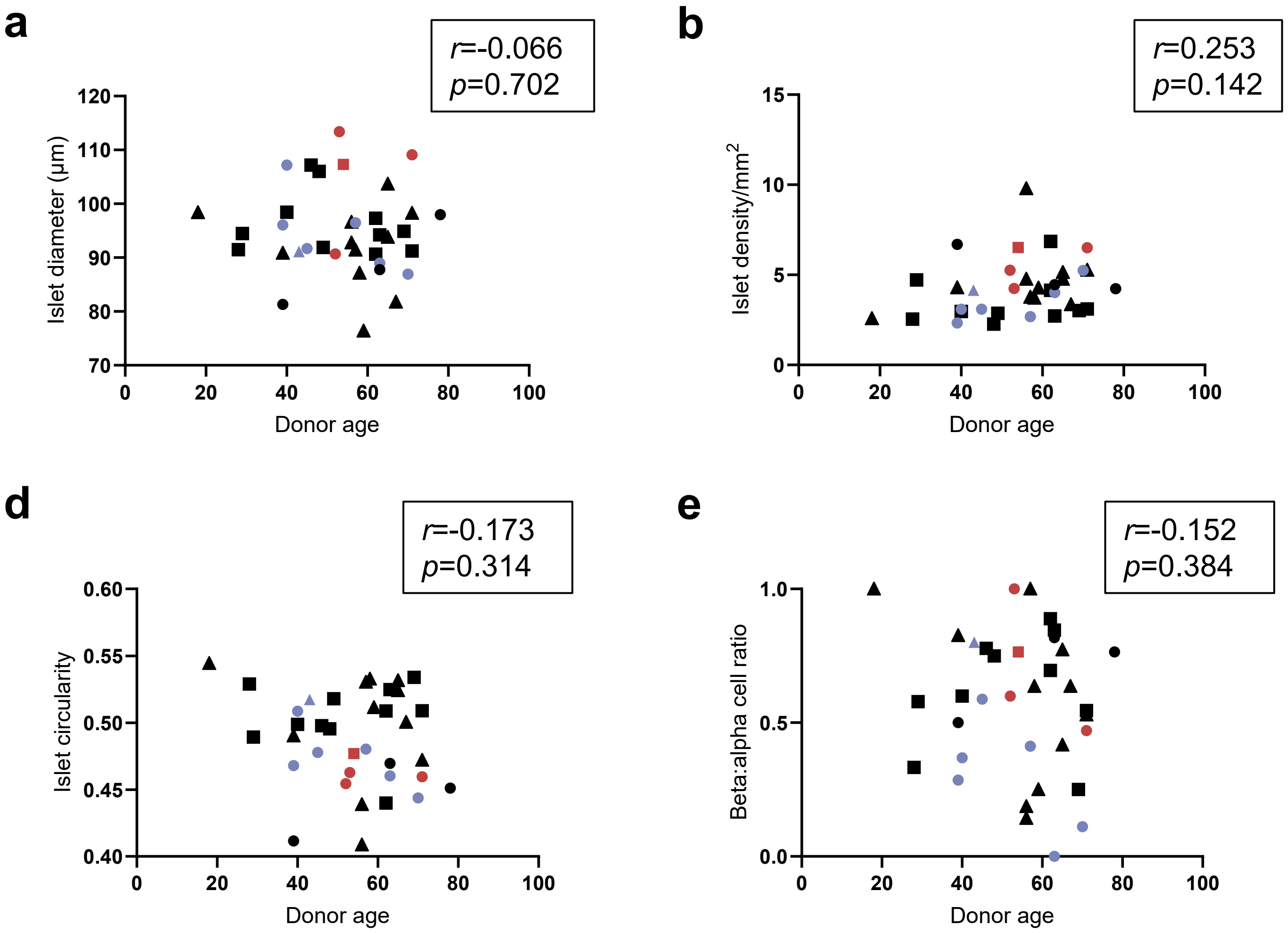

ESM Fig. 6. Islet morphometry versus donor age. (a): Islet diameter, (b): islet density, (c): endocrine pancreas mass, (d): islet circularity, and (e): beta:alpha cell ratio plotted against donor age. *r*: Pearson's correlation coefficient. n=33 (c), n=35 (b, e), n=36 (a, d). ND, no history of diabetes; T2D, type 2 diabetes.

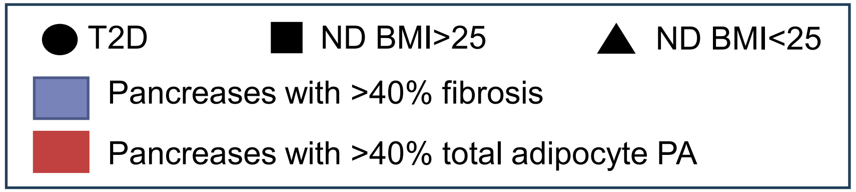

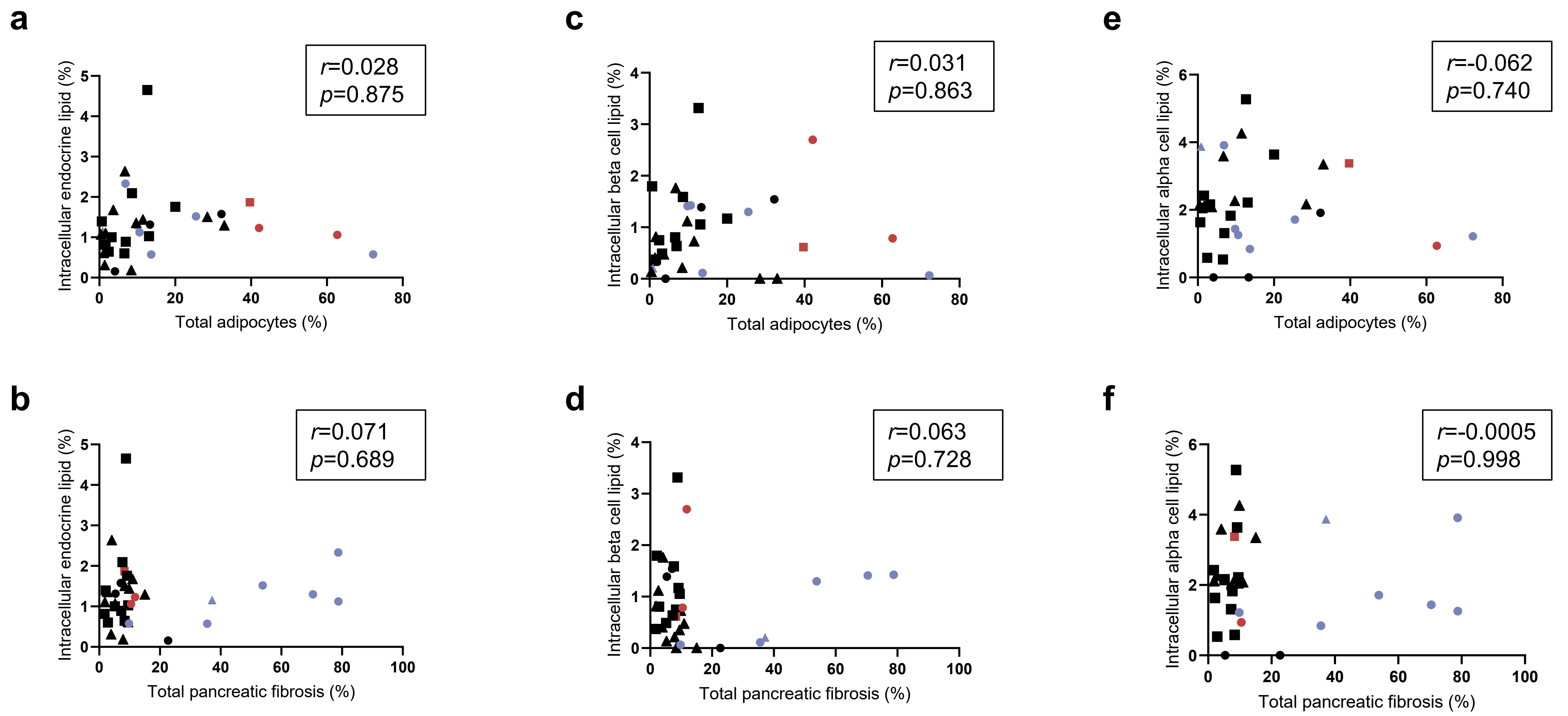

ESM Fig. 7. Relationship between intracellular endocrine lipid, adipocyte and fibrosis PA. Intracellular endocrine (a, b), beta cell (c, d), and alpha cell (e, f) lipid plotted against (a, c, e): total adipocyte PA, and (b, d, f) total pancreatic fibrosis PA. *r*: Pearson's correlation coefficient. n=31 (e, f), n=33 (c, d), n=34 (a, b). ND, no history of diabetes; T2D, type 2 diabetes.

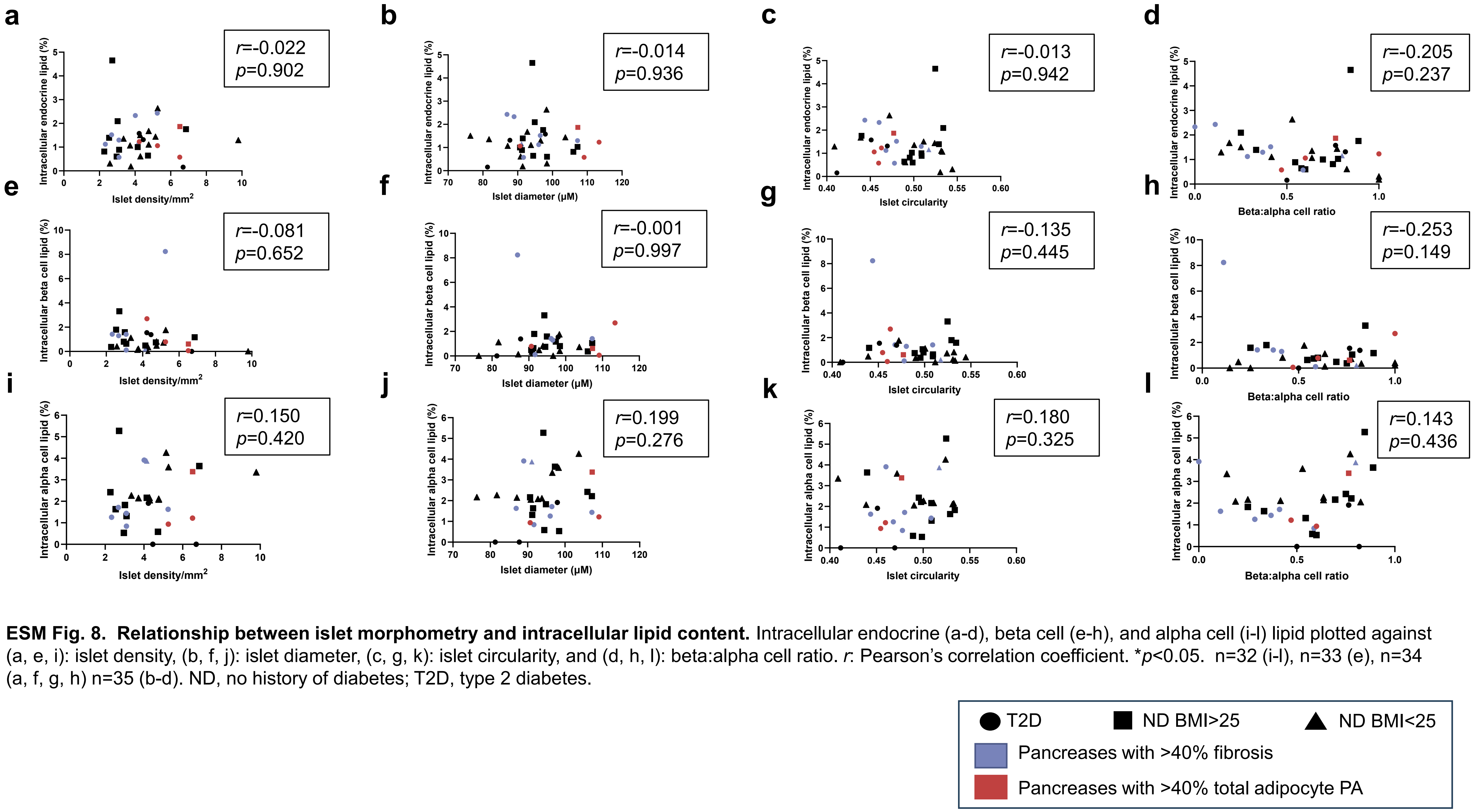

ESM Fig. 8. Relationship between islet morphometry and intracellular lipid content. Intracellular endocrine (a-d), beta cell (e-h), and alpha cell (i-l) lipid plotted against (a, e, i): islet density, (b, f, j): islet diameter, (c, g, k): islet circularity, and (d, h, l): beta:alpha cell ratio. *r*: Pearson's correlation coefficient. **p*<0.05. n=32 (i-l), n=33 (e), n=34 (a, f, g, h) n=35 (b-d). ND, no history of diabetes; T2D, type 2 diabetes.

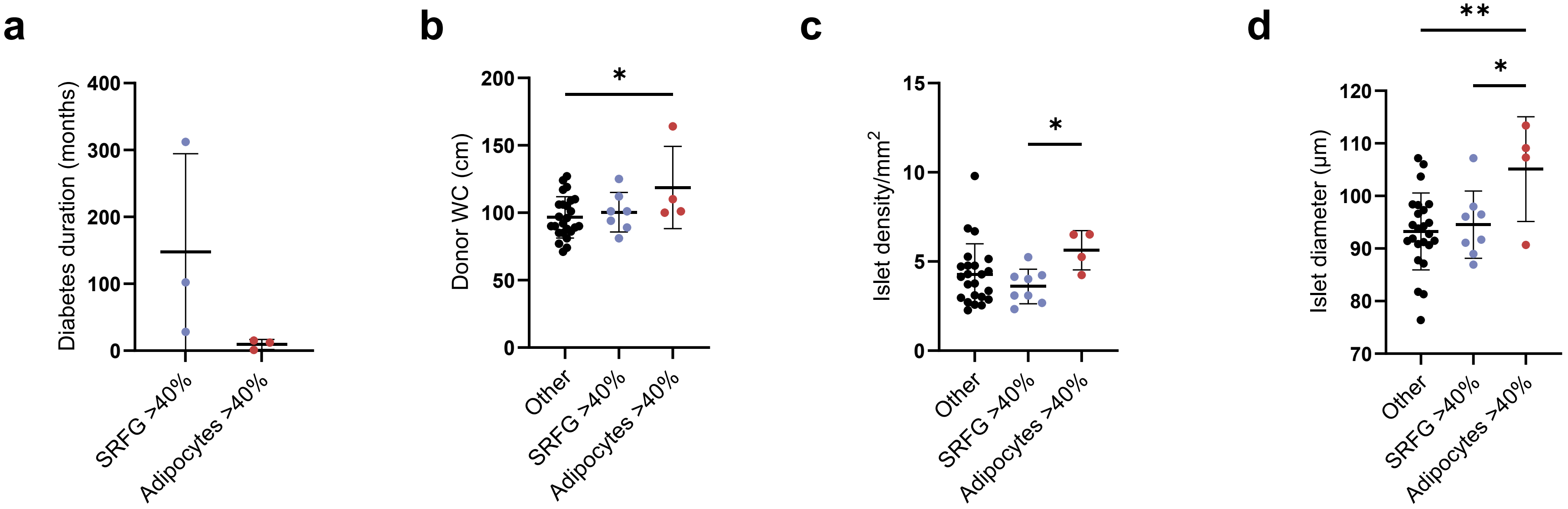

ESM Fig. 9. Diabetes duration, donor WC, islet density and islet diameter in 'high-fat' and 'high-fibrosis' donors. (a): Diabetes duration, (b): donor waist circumference, (c): islet density, (d): islet diameter by 'high-fat'/'high-fibrosis' phenotype. Bars indicate mean +/-SD. **p*<0.05, ***p*<0.01. n=9 (a), n=35 (c), n=36 (b, d).

Pancreases with >40% fibrosis

Pancreases with >40% total adipocyte PA

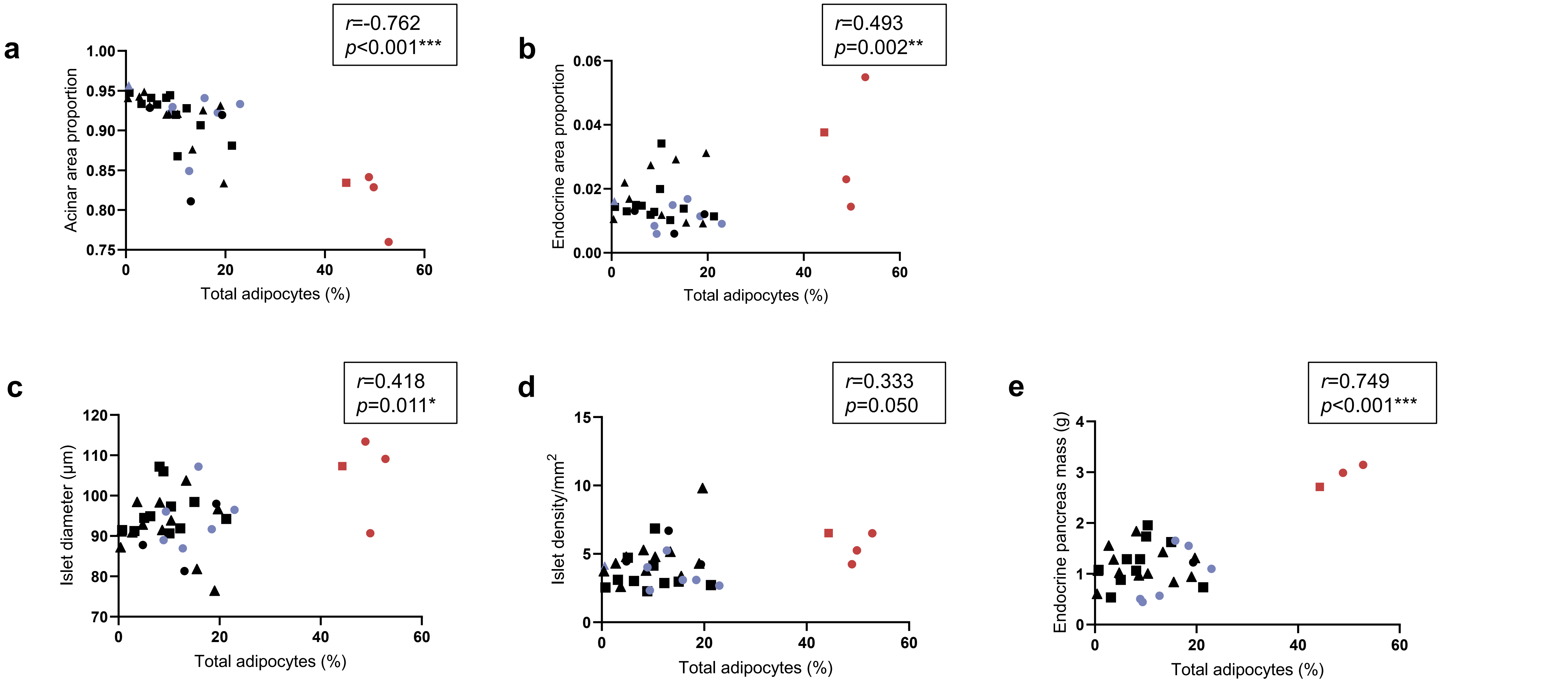

ESM Fig. 10. Correlation of adipocytes with acinar/endocrine proportional area and islet morphometry.
Total adipocytes plotted against (a): acinar area proportion, (b): endocrine area proportion, (c): islet diameter, (d): islet density, (e): endocrine pancreas mass. *r*: Pearson's correlation coefficient. *$p<0.05$, **$p<0.01$, ***$p<0.001$. n=33 (e), n=35 (c), n=36 (all other figures). ND, no history of diabetes; T2D, type 2 diabetes.

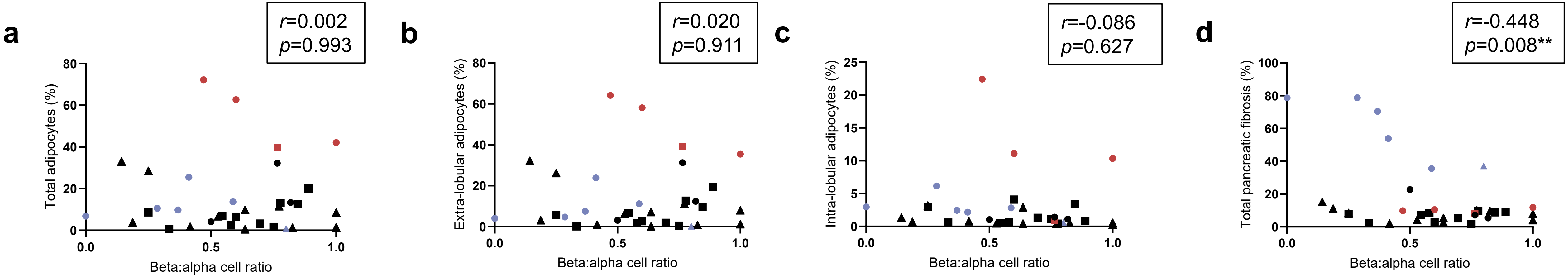

ESM Fig. 11. Pancreatic endocrine beta:alpha-cell ratios.
Beta:alpha cell ratio plotted against (a): total adipocytes, (b): extra-lobular adipocytes, (c): intra-lobular adipocytes, (d): total pancreatic fibrosis. Correlations were determined using the P4 pancreas region alone. *r*: Pearson's correlation coefficient. ***p*<0.01. n=34. ND, no history of diabetes; T2D, type 2 diabetes.

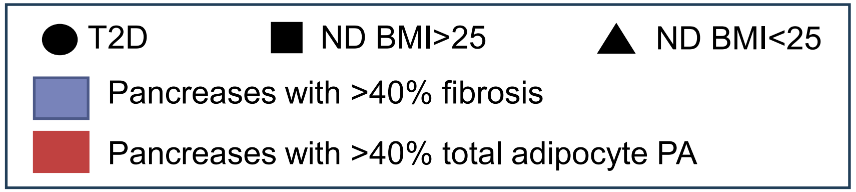

Supplement: Supplementary file 1 — ESM (PDF 849 KB) [file 125_2025_6547_MOESM1_ESM.pdf]
